# Middle East respiratory syndrome coronavirus: quantification of the extent of the epidemic, surveillance biases, and transmissibility

**DOI:** 10.1016/S1473-3099(13)70304-9

**Published:** 2014-01

**Authors:** Simon Cauchemez, Christophe Fraser, Maria D Van Kerkhove, Christl A Donnelly, Steven Riley, Andrew Rambaut, Vincent Enouf, Sylvie van der Werf, Neil M Ferguson

**Affiliations:** a MRC Centre for Outbreak Analysis and Modelling, Department of Infectious Disease Epidemiology, Imperial College London, London, UK; b Institute of Evolutionary Biology, Ashworth Laboratories, University of Edinburgh, Edinburgh, UK; c Institut Pasteur, Unit of Molecular Genetics of RNA Viruses, UMR3569 CNRS, Université Paris Diderot Sorbonne Paris Cité, Paris, France

## Abstract

**Background:**

The novel Middle East respiratory syndrome coronavirus (MERS-CoV) had, as of Aug 8, 2013, caused 111 virologically confirmed or probable human cases of infection worldwide. We analysed epidemiological and genetic data to assess the extent of human infection, the performance of case detection, and the transmission potential of MERS-CoV with and without control measures.

**Methods:**

We assembled a comprehensive database of all confirmed and probable cases from public sources and estimated the incubation period and generation time from case cluster data. Using data of numbers of visitors to the Middle East and their duration of stay, we estimated the number of symptomatic cases in the Middle East. We did independent analyses, looking at the growth in incident clusters, the growth in viral population, the reproduction number of cluster index cases, and cluster sizes to characterise the dynamical properties of the epidemic and the transmission scenario.

**Findings:**

The estimated number of symptomatic cases up to Aug 8, 2013, is 940 (95% CI 290–2200), indicating that at least 62% of human symptomatic cases have not been detected. We find that the case-fatality ratio of primary cases detected via routine surveillance (74%; 95% CI 49–91) is biased upwards because of detection bias; the case-fatality ratio of secondary cases was 20% (7–42). Detection of milder cases (or clinical management) seemed to have improved in recent months. Analysis of human clusters indicated that chains of transmission were not self-sustaining when infection control was implemented, but that *R* in the absence of controls was in the range 0·8–1·3. Three independent data sources provide evidence that *R* cannot be much above 1, with an upper bound of 1·2–1·5.

**Interpretation:**

By showing that a slowly growing epidemic is underway either in human beings or in an animal reservoir, quantification of uncertainty in transmissibility estimates, and provision of the first estimates of the scale of the epidemic and extent of case detection biases, we provide valuable information for more informed risk assessment.

**Funding:**

Medical Research Council, Bill & Melinda Gates Foundation, EU FP7, and National Institute of General Medical Sciences.

## Introduction

The earliest known human infections with Middle East respiratory syndrome coronavirus (MERS-CoV) occurred in Jordan in March, 2012,^1^ with isolation and identification of the virus from a patient in Saudi Arabia occurring some months later.^2^ By Aug 8, 2013, 94 virologically confirmed human cases and 17 probable cases^1^, ^3^, ^4^, ^5^ had been reported. Zoonotic exposure is suspected as the source of human infection, in view of the initially sporadic occurrence of cases together with the genetic similarity of MERS-CoV to bat coronaviruses.^6^ However, epidemiological investigations of cases did not find a consistent pattern of exposure to animals or the environment,^7^ and, as of Aug 8, 2013, the virus had not been isolated from any animal species. Recently, however, camels from Oman and the Spanish Canary Islands were discovered to have been previously infected by the MERS-CoV or a closely related virus.^8^ The high apparent case-fatality ratio in reported cases is cause for concern: as of Aug 8, 2013, WHO reported that 46 of the 94 virologically confirmed cases had died;^7^ this death toll is expected to rise since some patients are still in hospital.^9^

Although progress has been made in characterising the epidemiology of MERS-CoV, many uncertainties remain.^10^ Little is known about the extent of human infection or the degree of detection bias towards more severe cases. If the severe cases currently being detected represent only a small sentinel minority of a much larger number of milder cases (as occurred early in the 2009 H1N1 pandemic in Mexico^11^), the case-fatality ratio might be substantially lower than what current surveillance data suggest. Conversely, for the severe acute respiratory syndrome (SARS) epidemic of 2003, little evidence of undetected mild or subclinical infections existed,^12^ even after detailed serological follow-up studies.^13^ In the absence of robust community-based serological surveys for MERS-CoV, assessment of the extent of undetected infection must rely on epidemiological inference. Another essential aspect of risk assessment is the characterisation of transmissibility, and a study^14^ concluded that transmission levels were under the epidemic threshold in detected clusters.^14^ However, since stringent control measures were implemented in many of those clusters, the transmissibility in the absence of such controls is still unknown.

Here, we undertook analyses of epidemiological and genetic data to assess the number of cases missed by surveillance, the detection bias towards severe cases, and the transmission potential of MERS-CoV with and without control measures. We also estimated risk factors for fatal disease outcomes.

## Methods

### Data, incubation times, and incidence calculations

The appendix shows further details of all methods used. We assembled a comprehensive database of all confirmed and probable cases up to Aug 8, 2013, from public sources (appendix). We classified cases with known epidemiological links as a cluster, with singleton cases being viewed as independent clusters of size one.

We estimated the incubation period from travel-related clusters. In the absence of other potential exposures, we extracted detailed information on the exposure of secondary cases to the index case from the medical literature (appendix). A lognormal distribution was fitted to the data.

We obtained an approximate lower bound for the generation time *T_G_* (mean time from symptom onset of a case to symptom onset of their secondary cases) by considering the average delay between onset of first and second cases in clusters with more than one case. A Gamma distribution was fitted to the data.

Since discovery of an index case prompts enhanced surveillance in contacts of that case, the occurrence of detected clusters is likely to represent a more reliable indication of changes in the underlying population incidence than the incidence of cases. We therefore used observed cluster incidence over time to study growth in incidence over time. An exponential growth model was fitted to these data and the doubling time of the epidemic was estimated (appendix).

We obtained an independent estimate of doubling time by phylogenetic analysis of seven sequences from different MERS-CoV case clusters (appendix). We applied Bayesian phylogenetic and coalescent models assuming a strict molecular clock and exponential growth of the viral population (appendix).

Using data of numbers of visitors to Jordan, Qatar, Saudi Arabia, and United Arab Emirates who are resident outside the Middle East (denoted returning non-resident travellers) and their duration of stays, we estimated the expected number of symptomatic cases among residents in those countries from the incidence in travellers under the assumption that residents and visitors had an equal per-day risk of infection (appendix).

From estimates of the growth rate of the viral population derived from analysis of available MERS-CoV genetic sequences, we also estimated the total number of infections that occurred between the time to most recent common ancestor (TMRCA) and Aug 8, 2013.

### Assessment of the transmission scenarios

We used the size of detected clusters to estimate the reproduction number averaged across all cases in detected clusters (*R_cluster_*).

Classifying the case with the earliest onset date in every cluster as the index case, we also inferred the numbers of secondary cases infected by the index case (*R_index_*) probabilistically using the generation time distribution and timing of cases (appendix).^15^

We also fitted a model of animal-to-human and human-to-human transmission to the incidence of clusters over time, and the cumulative incidence of returning non-resident traveller cases, for different values of *R* (appendix). We modelled infection in an animal species that was assumed to seed infections into man. Animals infected other animals, whereas human beings could be infected by animals or other human beings.

### Role of funding source

The sponsor of the study had no role in study design, data collection, data analysis, data interpretation, or writing of the report. The corresponding author had full access to all the data in the study and had final responsibility for the decision to submit for publication.

## Results

**Figure 1:**
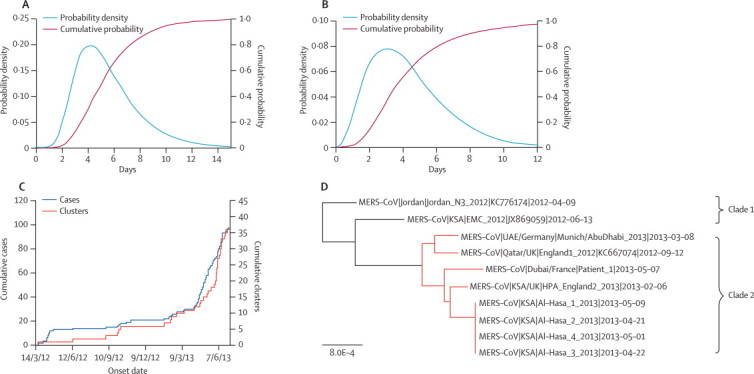
Epidemiological and genetic data (A) Probability density and cumulative probability of the incubation period from data of exposure for a subset of seven cases. (B) Probability density and cumulative probability of the delay between onset of first and second cases in six case clusters with more than one case. The delay between onset of first and second cases is a lower bound of the generation time. (C) Cumulative number of confirmed cases and clusters detected of MERS-CoV. (D) Maximum likelihood phylogeny of viral sequences of MERS-CoV, obtained using PhyML with the TN93 model. More recent samples were found to cluster together (highlighted in red), suggesting these viruses are part of an emerging epidemic. Only Al-Hasa_1 was included in analysis, to avoid over-representation of this outbreak.

Cases detected in the UK, France, Italy, and Tunisia caused seven secondary cases in those countries (appendix). We estimated the mean of the incubation period distribution as 5·5 (95% CI 3·6–10·2) and the SD as 2·5 (95% CI 1·2–11·6) days (figure 1A), compatible with results from a study by Assiri and colleagues.^3^ This finding suggests the mean generation time, *T_G_*, is unlikely to be shorter than 7 days.

For the six clusters (in France, Jordan, Saudi Arabia, and the UK) for which full data on symptom onset dates are available, the lower bound for *T_G_* has a mean of 10·7 (95% CI 6·5–19·4) days and an SD of 6·3 (95% CI 3·5–16·9) days (figure 1B). In the following, we therefore explore values of *T_G_* in the range 7–12 days, while fixing the coefficient of variation of the generation time at 0·45, based on estimates found for SARS.^16^

From the distribution of cluster sizes (average 2·7; range 1–26), and consistent with a study by Breban and colleagues,^14^ we estimated that *R* averaged across all cases in detected clusters (*R_cluster_*) was 0·63 (0·47–0·85). We also found a trend for a reduction of cluster size with time: 11 of 21 (52%; 95% CI 30–74) clusters were single case clusters before June 1, 2013, as opposed to 16 of 19 (84%, 60–97) after June 1, 2013 (p=0·07), leading to *R_cluster_* dropping from 0·74 (95% CI 0·53–1·03) before June 1, 2013 to 0·32 (95% CI 0·14–0·65) after June 1, 2013. These changes could be explained by faster detection of cases and introduction of controls: the median delay from first onset in the cluster to reporting of the cluster moved from 23 days before June 1, 2013 (n=14) to 8 days (n=4) after June 1, 2013. The estimate of *R_cluster_* indicates that transmission is not self-sustaining in man once control measures are in place.

The reproduction number of cluster index cases (*R_index_*) was expected to be less affected by control measures because of the delay between onset in the index case to the detection of the case and implementation of controls. This was particularly the case before June 1, 2013, when 93% (13 of 14) of delays from onset to reporting of cluster were 16 days or longer. Analysis of clusters with complete symptom information on this time period gave *R_index_*=1·25 (95% CI 1·00–1·50) for *T_G_* =12 days and *R_index_*=0·83 (95% CI 0·67–1·08) for *T_G_* =7 days. A sensitivity analysis gave central estimates in the range 0·8–1·1 (appendix).

Figure 1C presents temporal trends in infection incidence using epidemiological data on reported cases and clusters. The analysis of observed cluster incidence over time indicates a doubling time of 90 (95% CI 65–133) days.

The phylogeny showed substantial diversity, with sequences obtained from September, 2012, onwards falling into a single clade, and two older sequences forming a distinct outgroup (figure 1D). The TMRCA for all seven sequences was June 27, 2011 (95% CI Sept 25, 2010–Nov 8, 2011), whereas that for the recent clade of five isolates (clade 2) was June 11, 2012 (April 24, 2012–July 18, 2012). The independent estimate of doubling time of the viral population in the recent clade (clade 2) is 41 days (95% CI 17–234).

Using a best estimate of a mean 4 day stay for returning non-resident traveller cases (appendix), we estimated a total of 940 (95% CI 290–2200) symptomatic cases occurred in residents of these countries up to the end of Aug 8, 2013.

Using available MERS-CoV genetic sequences the median estimate of cumulative infections (in man and in the reservoir) that occurred between June, 2012 (the TMRCA of the recent clade), and Aug 8, 2013, is 5640 (IQR 780–55 430). In deriving these estimates, we assumed a single generation time distribution, meaning the estimates were valid either if human-to-human transmission was self-sustaining and human infections therefore predominated, or if generation times in any animal reservoir were similar to those in man—in which case the estimates represented total infections in man and the animal reservoir.

On its own, a purely genetic analysis with few sequences can say little about the species within which sequence evolution is occurring, and, as a result, different scenarios for MERS-CoV are possible. The two older sequences could represent independent introductions of the virus from an animal reservoir, whereas the more recent clade could represent sustained human-to-human transmission. Alternatively, the growth could be generated by an epidemic in an animal reservoir, with associated increasing incidence of spill-over infections in human beings.

Irrespective of the scenario, we can use estimates of doubling times to derive an upper bound for *R*. Under the assumption that growth outside detected clusters is driven by human-to-human transmission, *R* would remain close to 1: for *T_G_* =12, *R*=1·21 (95% CI 1·02–1·53) from an analysis of the five more recent sequences and *R*=1·10 (95% CI 1·06–1·13) from the timing of incident clusters; for *T_G_*=7, *R*=1·12 (95% CI 1·01–1·29) from an analysis of the five more recent sequences and *R*=1·06 (95% CI 1·04–1·08) from the timing of incident clusters. We obtained similar estimates from the timing of incident cases (appendix).

**Figure 2:**
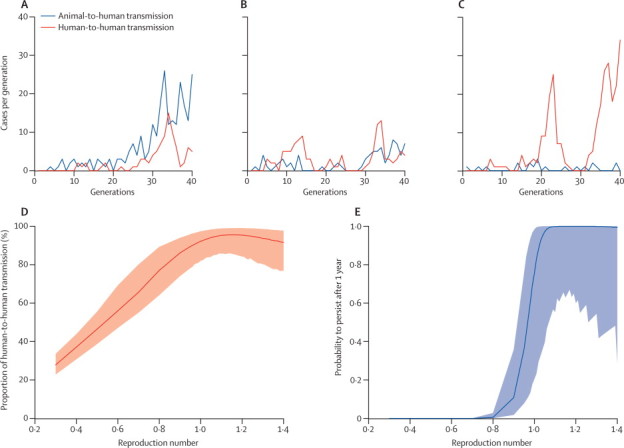
Alternative scenarios for animal-to-human and human-to-human transmission (A–C) Illustrative epidemic trajectories (incidence of human infections occurring in each transmission generation of length *T_G_*=12 days) consistent with the timing of clusters and data on returning non-resident traveller cases for *R*_0_=0·3 (A), *R*_0_=0·7 (B), and *R*_0_=1·06 (C). (D) Proportion of human cases due to human-to-human transmission in the epidemic so far as a function of the reproduction number, for *T_G_*=12 days. (E) Probability that current chains of transmission will be sustained for a finite period (1 year) as a function of the reproduction number, for *T_G_*=12 days. See appendix for details.

Figure 2 shows possible epidemic trajectories of animal-to-human and human-to-human transmission for different values of *R* for *T_G_*=12 days. The proportion of human-to-human infections was about 30% for *R*=0·3 and close to 100% for *R* greater than 1 (figure 2D). Assessment of the probability that current chains of transmission will be sustained into the future (figure 2E, appendix) showed that if *R* was greater than 1, then human-to-human transmission was likely to have already reached a degree in which extinction by chance was improbable. The analysis also confirmed that *R* was small (*R*≤1·17 for *T_G_*=12 and *R*≤1·10 for *T_G_*=7 days; appendix), but could not discriminate robustly between scenarios in which the virus was self-sustaining in man and scenarios in which it was not.

Our conclusion that case ascertainment had been low to date also received some support from analysis of the line list (appendix). Routine surveillance is likely to be biased towards the detection of more severe cases so that the case-fatality ratio estimated from these cases might be a substantial overestimate. We expect secondary cases detected via enhanced surveillance of case contacts to constitute a more representative sample of cases in general. Of those patients with a known outcome (fatal or recovered), 14 of 19 (74%; 95% CI 49–91) patients with infection detected through routine surveillance and five of 24 (21%; 7–42) patients representing secondary cases died (relative risk [RR]=3·54; 95% CI 1·55–8·07; p=0·003).

In patients with a known outcome, the probability of a fatal outcome was 77% (27 of 35; 95% CI 60–90) for those older than the median case age of 50 years; but only 22% (nine of 40; 11–38) for those younger than 50 years (relative risk RR 3·43; 95% CI 1·88–6·26; p=0·0001). In the 56 patients with data about age and about whether they were the first detected case of a cluster, 20 of 23 (87%; 95% CI 66–97) of those aged older than 50 years were the first detected cases as opposed to 11 of 33 cases younger than 50 years (33%; 18–52; RR=2·61; 95% CI 1·57–4·33; p=0·0002). This result was consistent with the hypothesis that cases in older patients were more likely to be severe, and that severe cases were more likely to be detected through routine surveillance.

**Figure 3:**
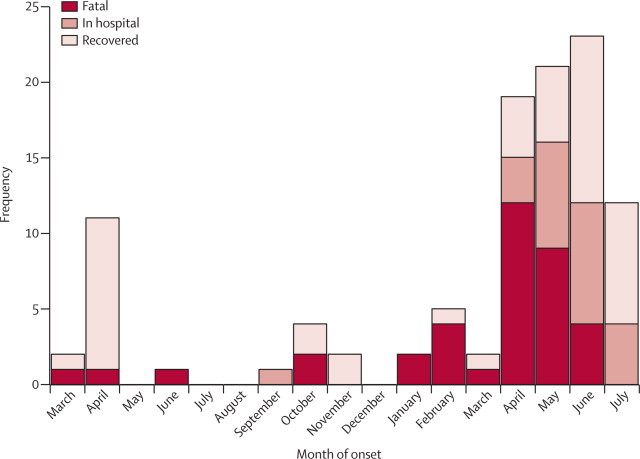
Outcome as a function of month of symptom onset for years 2012–13 When onset information was missing, the date of symptom onset was estimated by subtracting 10 days (the median delay from onset to reporting in 2013, once the Al Hasa cluster had been excluded) to the date of reporting.

Since June, 2013, we have noted a trend for cases being less likely to be fatal (figure 3), but this trend cannot be fully resolved yet because of the uncertainty about those patients who are still in hospital. However, we noted that the difference would remain significant even if 70% of those in hospital died (RR 1·71; 95% CI 1·04–2·81; p=0·036). This suggests detection of milder cases (or clinical case management) has improved with time.

## Discussion

In this study, we analysed publicly available epidemiological and genetic data to evaluate the extent of human infection, the performance of case detection, and the transmission potential of MERS-CoV with and without control measures (panel).

Panel **Research in context**

**Systematic review**

MERS-CoV cases continue to be reported (150 confirmed cases at the time this Article was going to press on Nov 5, 2013), largely in Middle Eastern countries, but by far, the largest number of cases have been reported in Saudi Arabia. Evidence is growing that the MERS-CoV virus, or a closely related virus, has infected camels in Egypt and Qatar, and probably in many other countries throughout the affected region. Without finding the virus in animals, it is impossible to know the reservoir(s) of this virus and stop transmission from animals to man.

We analysed all publicly available epidemiological data up to Aug 8, 2013, provided by WHO^7^, ^17^ from 111 virologically confirmed or probable human MERS-CoV cases distributed over the eight affected countries and publicly available genetic data, assessed the extent of human infection, the performance of case detection, and the transmission potential of MERS-CoV with and without control measures. A PubMed search on Sept 24, 2013, with the terms “HCoV-EMC”, “MERS-CoV”, and “novel coronavirus” identified 27, 63, and 279 papers, respectively. All relevant papers are included in this report. We also searched WHO, and the Saudi Arabia Ministry of Health websites and reviewed all publications for MERS-CoV published to date.

**Interpretation**

Our report is the first to estimate the total number of symptomatic cases from returning non-resident traveller cases and to quantify detection biases towards severe cases. Breban and colleagues^14^ found that in clusters that were detected and investigated, the reproduction number was smaller than 1. Since control measures were implemented in many of these clusters, transmission in the absence of control measures remained unknown. We did independent analyses, looking at the growth in incident cases and incident clusters, the growth in viral population, and the reproduction number of cluster index cases to derive upper bounds of *R* in the absence of control measures.

We used data on returning non-resident traveller cases to estimate the total number of resident severe cases in the Middle East. By comparing this number to the number of reported cases in the Middle East, we conclude that at least 62% of clinically apparent cases have been missed. This conclusion is indirectly supported by our independent finding that detection of milder cases seems to have improved in recent months. Since the four returning non-resident traveller cases were clinically severe, this first analysis reflects missed severe clinical cases. This contrasts with the genetic analysis that estimates the number of all infections in both man and animals (albeit assuming the generation time of infection in the same in all species). The larger estimates obtained from the genetic analysis might therefore be explained by the presence of milder cases or, alternatively, by a substantial number of infections occurring in animals.

Implicit in the estimation of the cumulative number of cases from returning non-resident traveller cases is an assumption that visitor and resident populations have a similar composition in terms of age, sex, and prevalence of comorbidities that might increase either susceptibility to infection or the risk of severe disease. If their demographic composition or health characteristics put visitors at greater risk of severe disease than residents, then this approach might overestimate case numbers in the resident population. If the epidemic is driven by human-to-human transmission, it is plausible that visitors might have a lower risk of infection than locals (because of inhomogeneous mixing patterns). By contrast, in the alternative scenario of an animal reservoir such as camels,^8^ visitors could be at greater risk of infection if they were more likely to be in contact with the reservoir than locals. However, without better characterisation of risk factors for severe MERS-CoV disease and data on visitor characteristics, it is not currently possible to adjust for such potential biases.

Consistent with a previous study,^14^ our analysis of cluster sizes indicated that *R* averaged across all cases in detected clusters (*R_cluster_*) was smaller than 1. This finding indicates that chains of transmission are not self-sustaining when infection control measures are implemented. The reproduction number of index cases (*R_index_*), which is less likely to be affected by control measures, had central estimates in the range 0·8–1·3. So the scenario of *R* slightly above 1 in the absence of control measures cannot be ruled out from these data. We found that the size of clusters (and *R_cluster_*) decreased over time, which might be explained by shorter delays in onset to detection and control. Other possible explanations include seasonal variations in transmissibility or a reduction in the propensity to investigate clusters when cases are detected. Collecting more precise data about the timing and nature of infection control measures would reduce uncertainty about *R*.

Both the analysis of the genetic sequences and of the epidemic curves suggested that an epidemic is under-way either in an animal reservoir or in man. These analyses do not allow us to distinguish between these two scenarios and to determine whether MERS-CoV is currently self-sustaining in man. However, they can be used to derive an upper bound for *R* in the range 1·2–1·5, indicating that *R* cannot be much above 1. The growth in the rate at which clusters are detected could be explained by an increase in the reporting rate rather than a true increase in incidence rate (appendix). However, the genetic analysis, which is less sensitive to temporal variation in levels of case detection, suggests that the observed increased incidence of cases and clusters in 2013 is not just a reporting artifact.

A caveat to the estimation of *R_index_* is that there might be a surveillance bias towards detecting clusters which have an index case that causes multiple secondary cases. However, it is notable that each of the four reported cases in travellers caused one or two secondary cases (mean *R*=1·75) on their return to their country of residence, and yet detection of secondary cases occurred after the identification of infection in the index traveller. Transmission probabilities might be boosted in populations with comorbidities in which many of the clusters took place; on the other hand, *R_index_* might underestimate transmission in the absence of controls if interventions were promptly implemented after onset in the index case.

Cotten and colleagues^18^ recently published an analysis of a large number of sequences. Many of these sequences were from the Al-Hasa outbreak, and so although very interesting, do not add to the population dynamic inferences. During late revision stage, we repeated our analysis with these new sequences. Our analysis is most directly similar to their figure 3, an analysis of 11 representative sequences. We further removed two sequences (Al Hasa 8, so that the outbreak is only represented once, and Bisha-1 because of the controversy surrounding it and the encephalomyocarditis virus), and added the new Paris sequence. Based on this new dataset, we estimated the molecular clock rate at 1·0 × 10^−3^ substitutions per site per year with 95% credibility interval (6·8 × 10^−4^–1·3 × 10^−3^) that overlaps the estimates of Cotten and colleagues.^18^ Based on this updated analysis, the updated TMRCA for all sequences was Oct 23, 2010 (95% CI Nov 28, 2008–July 3, 2011), whereas that for the recent clade of now 11 isolates was March 29, 2012 (Nov 11, 2011–June 7, 2012). The independent estimate of doubling time of the viral population in the recent clade is 43 days (95% CI 23–104). Using available MERS-CoV genetic sequences the median estimate of cumulative infections (in man and in the reservoir) that occurred between March, 2012 (the TMRCA of the recent clade), and Aug 8, 2013, is 17 490 (IQR 3902–94 507).

We conclude that a slowly growing epidemic is underway, but current epidemiological data do not allow us to determine whether transmission is self-sustaining in man. Our analysis demonstrates that the transmissibility of MERS-CoV in man is close to the critical threshold of *R*=1 required for self-sustaining transmission. If *R* is greater than 1, then the number of human cases we estimate to have occurred to date make it highly likely that self-sustaining transmission has already begun. If a human epidemic is underway, the low estimated value of *R* (<1·3–1·5) and evidence of severe infection in secondary cases from case clusters suggest that intensive public health measures around cases, coupled with improved case ascertainment, are sufficient to contain spread and reduce morbidity and mortality. However, an important caveat to this conclusion is the unknown extent of mildly symptomatic or asymptomatic infection and the role it might have in transmission.^19^ Our analysis indicates a high proportion of infections are not currently being detected. The reported case data therefore probably represent the severe end of a wider clinical spectrum of disease, though a case-fatality ratio higher than 10% cannot be ruled out from present data. Paradoxically, a low case-fatality ratio might make control more difficult, if mildly symptomatic cases prove to be responsible for most transmission.

Improved surveillance, international collaboration, and data-sharing are therefore crucial to refining our understanding of the transmission dynamics and epidemiology of this novel human virus and of the risk it poses. The time window might be short for doing so: current selection pressures on the virus to evolve increased transmissibility in man are likely to be intense.^20^
