# Supplementary material for: Middle East respiratory syndrome coronavirus: quantification of the extent of the epidemic, surveillance biases, and transmissibility
Source: Lancet Infect Dis. 2014 Jan;14(1):50–6. doi: 10.1016/S1473-3099(13)70304-9 (PMC3895322; doi:10.1016/S1473-3099(13)70304-9)
Supplement: Supplementary appendix [file mmc1.pdf]

THE LANCET Infectious Diseases

## Supplementary webappendix

This webappendix formed part of the original submission and has been peer reviewed. We post it as supplied by the authors.

Supplement to: Cauchemez S, Fraser C, Van Kerkhove MD, et al. Middle East respiratory syndrome coronavirus: quantification of the extent of the epidemic, surveillance biases, and transmissibility. *Lancet Infect Dis* 2013; published online Nov 13. http://dx.doi.org/10.1016/S1473-3099(13)70304-9.

*Supplementary Material*

# Middle East respiratory syndrome coronavirus: Quantifying the extent of the epidemic, surveillance biases and transmissibility thus far

**Simon Cauchemez[1]*, Christophe Fraser[1]*, Maria D. Van Kerkhove[1], Christl A. Donnelly[1], Steven Riley[1], Andrew Rambaut[2], Vincent Enouf[3], Sylvie van der Werf[3], Neil M. Ferguson[1#]**

*contributed equally

[#] to whom correspondence should be addressed (neil.ferguson@imperial.ac.uk)

1: MRC Centre for Outbreak Analysis and Modelling, Department of Infectious Disease Epidemiology, Imperial College London, London, UK (S Cauchemez PhD, Prof C Fraser PhD, MD Van Kerkhove PhD, Prof CA Donnelly ScD, S Riley PhD, Prof NM Ferguson DPhil)

2: Institute of Evolutionary Biology, Ashworth Laboratories, University of Edinburgh, Edinburgh, UK (Prof A Rambaut PhD)

3: Institut Pasteur, Unit of Molecular Genetics of RNA Viruses, UMR3569 CNRS, Université Paris Diderot Sorbonne Paris Cité, Paris, France (V Enouf PhD, Prof S van der Werf PhD)

**Contents**

1 Epidemiologic data ........ 3

2 Incubation period ........ 8

3 Delay between onsets in the first 1st and 2nd case in clusters ........ 8

4 Epidemic curve, exponential growth and reproduction number ........ 9

5 Reproduction number averaged across all cases in the cluster ($R_{cluster}$) ........ 12

6 Reproduction number of the index case in clusters ($R_{index}$) ........ 13

7 Using cases among returning non-resident travellers to infer comparable domestic case numbers ........ 14

8 Balance between animal-to-human and human-to-human transmission ........ 18

9 Summary of genetic analysis of MERS-CoV ........ 22

9.1 The data ........ 22

9.2 Choosing the substitution model for phylogenetic analysis ........ 22

9.3 Maximum likelihood phylogeny ........ 23

9.4 Evidence for a molecular clock ........ 23

9.5 Bayesian coalescent inference of population dynamics ........ 26

9.5.1 Methods ........ 26

9.5.2 Dated phylogeny ........ 26

9.5.3 Population dynamics ........ 26

9.5.4 Number of infected individuals ........ 27

9.6 Sensitivity analyses ........ 28

9.6.1 Sensitivity analysis to the choice of rooting ........ 28

9.6.2 Sensitivity to choice of clock rate ........ 29

9.6.3 Sensitivity to choice of generation time distribution ........ 30

9.6.4 Table S15. Sensitivity of genetic estimates to mean generation time, $T_g$ , keeping coefficient of variation of the generation time distribution constant. ........ 30

*9.6.5* Sensitivity to choice of dispersion parameter $k$ ........ 31

9.6.6 Sensitivity to choice of substitution model ........ 31

9.7 Update of the phylogenetic analysis, added in proof ........ 32

10 References ........ 37

# 1 Epidemiologic data

As of 8 August 2013, 94 laboratory-confirmed cases of MERS-CoV were reported to the World Health Organization (WHO) many of whom have been described in either case or cluster reports [1-22]. A line list was compiled using publically reported data on laboratory-confirmed MERS-CoV cases from a number of sources including WHO, ProMed and in the peer-reviewed literature [1-22]. An additional 17 probable cases have been reported from investigations [6, 8, 21, 23]. Using these sources, the following data were extracted into the line list shown in Table S1; reporting date either by WHO or to ProMed, symptom onset, age, gender, country where case was identified, nationality and sub-nationality of case, location where infection is believed to have occurred, whether the case was exported to a country outside of the Middle East, country where case was treated, severity of disease, patient outcome, exposure information (i.e., recent travel history to the Middle East and duration of travel, reported animal exposures, if case was a health care worker), co-morbid conditions, and genetic sequencing resulted from any patient samples. A cluster identification number was assigned to cases with specific reported epidemiologic-links and to those reported as being part of clusters.

We define the “index case” as the case with earliest symptom onset in the cluster. The “primary case” is the first case detected in the cluster (not necessarily the index case).

## Table S1. Line List of Confirmed Cases as of 8 August 2013.

| Cluster ID | Date reported | Symptom onset date | age | sex | Country identified | Nationality | Sub-national | Location where infection is believed to have occurred | Exported case | Country where treated | Severity | Outcome | Recent travel history | Duration of travel | Animal Exposure | HCW | Co-morbid conditions | Source |
|---|---|---|---|---|---|---|---|---|---|---|---|---|---|---|---|---|---|---|
| 1 | 20/09/12 | 13/06/12 | 60 | m | KSA | KSA | Bisha | Bisha | no | KSA | severe | fatal | no | --- | NR | no | no | 14, 15 |
| 2 | 01/11/12 | 05/10/12 | 70 | m | KSA | KSA | Riyadh | Riyadh | no | KSA | severe | fatal | no | --- | NR | no | yes | 7, 8, 14 |
| 2 | 28/11/12 | 24/10/12 | 39 | m | KSA | KSA | Riyadh | Riyadh | no | KSA | severe | fatal | no | --- | NR | no | yes | 7, 8, 14 |
| 2 | 19/11/12 | 04/11/12 | 31 | m | KSA | KSA | Riyadh | Riyadh | no | KSA | severe | recovered | no | --- | NR | no | no | 7, 8, 14 |
| 3 | 04/11/12 | 09/10/12 | 49 | m | KSA | KSA | Riyadh | Riyadh | no | KSA | severe | recovered | no | -- | yes | no | yes | 1, 14, 17 |
| 4 | 30/11/12 | 21/03/12 | 25 | m | Jordan | Jordan | Zarqa | Zarqa | no | Jordan | severe | fatal | no | -- | no | yes | NR | 6 |
| 4 | 30/11/12 | 02/04/12 | 40 | f | Jordan | Jordan | Zarqa | Zarqa | no | Jordan | severe | fatal | no | -- | no | yes | NR | 6 |
| 5 | 22/09/12 | 03/09/12 | 49 | m | UK | Qatar | Doha, Qatar | Doha, Qatar | no | Doha, UK | severe | in hospital | Saudi Arabia | NR | NR | no | NR | 2, 9, 14 |
| 6 | 01/11/12 | 01/10/12 | NR | m | Germany | Qatar | Doha, Qatar | Doha, Qatar | no | Germany | severe | recovered | yes | Qatar | yes | no | NR | 3, 14 |
| 7 | 11/02/13 | 24/01/13 | 60 | m | UK | UK | Birmingham | Riyadh | yes | UK | severe | fatal | Saudi Arabia, Pakistan | unknown | no | no | yes | 10-14 |
| 7 | 15/02/13 | 05/02/13 | 30 | f | UK | UK | Birmingham | Birmingham | no | none | mild | recovered | no | -- | no | no | no | 10-14 |
| 7 | 13/02/13 | 06/02/13 | 38 | m | UK | UK | Birmingham | Birmingham | no | UK | severe | fatal | no | -- | no | no | yes | 10-14 |
| 8 | 21/02/13 | NR | 61 | f | KSA | KSA | Riyadh | Riyadh | no | KSA | severe | fatal | no | -- | NR | no | no | 14 |
| 9 | 07/03/13 | NR | 65 | m | KSA | KSA | Bur Qassim | Bur Qassim | no | KSA | severe | fatal | no | -- | NR | no | no | 14 |
| 10 | 12/03/13 | 24/02/13 | 37 | m | KSA | KSA | Riyadh | Riyadh | no | KSA | severe | fatal | no | -- | NR | no | no | 14 |
| 10 | 23/03/13 | NR | NR | NR | KSA | KSA | NR | NR | no | KSA | mild | recovered | NR | -- | NR | no | NR | 14 |
| 11 | 26/03/13 | NR | 73 | m | Germany | UAE | Abu Dhabi | Abu Dhabi | no | Germany | severe | fatal | NR | -- | NR | no | NR | 14 |
| 12 | 19/06/13 | 09/04/13 | 58 | m | KSA | NR | Al Hasa | Al Hasa | no | KSA | severe? | recovered | no | --- | NR | no | NR | 21 |
| 12 | 19/06/13 | 14/04/13 | 59 | m | KSA | NR | Al Hasa | Al Hasa | no | KSA | severe | fatal | no | --- | NR | no | yes | 21 |
| 12 | 19/06/13 | 15/04/13 | 24 | m | KSA | NR | Al Hasa | Al Hasa | no | KSA | severe | in hospital | no | --- | NR | no | yes | 21 |
| 12 | 19/06/13 | 16/04/13 | 87 | m | KSA | NR | Al Hasa | Al Hasa | no | KSA | severe | fatal | no | --- | NR | no | yes | 21 |
| 12 | 19/06/13 | 18/04/13 | 77 | m | KSA | NR | Al Hasa | Al Hasa | no | KSA | severe | fatal | no | --- | NR | no | yes | 21 |
| 12 | 19/06/13 | 19/04/13 | 58 | m | KSA | NR | Al Hasa | Al Hasa | no | KSA | severe | in hospital | no | --- | NR | no | yes | 21 |
| 12 | 19/06/13 | 19/04/13 | 62 | f | KSA | NR | Al Hasa | Al Hasa | no | KSA | severe | in hospital | no | --- | NR | no | yes | 21 |
| 12 | 19/06/13 | 19/04/13 | 94 | m | KSA | NR | Al Hasa | Al Hasa | no | KSA | severe | fatal | no | --- | NR | no | NR | 21 |
| 12 | 19/06/13 | 20/04/13 | 56 | m | KSA | NR | Al Hasa | Al Hasa | no | KSA | severe | fatal | no | --- | NR | no | yes | 21 |
| 12 | 19/06/13 | 22/04/13 | 56 | m | KSA | NR | Al Hasa | Al Hasa | no | KSA | severe | fatal | no | --- | NR | no | yes | 21 |
| 12 | 19/06/13 | 23/04/13 | 50 | m | KSA | NR | Al Hasa | Al Hasa | no | KSA | mild? | recovered | no | --- | NR | no | NR | 21 |
| 12 | 19/06/13 | 25/04/13 | 52 | f | KSA | NR | Al Hasa | Al Hasa | no | KSA | severe | fatal | no | --- | NR | no | NR | 21 |
| 12 | 19/06/13 | 27/04/13 | 33 | f | KSA | NR | Al Hasa | Al Hasa | no | KSA | severe | recovered | no | --- | NR | no | NR | 21 |
| 12 | 19/06/13 | 28/04/13 | 81 | f | KSA | NR | Al Hasa | Al Hasa | no | KSA | severe | fatal | no | --- | NR | no | NR | 21 |

| | | | | | | | | | | | | | | | | | | |
|---|---|---|---|---|---|---|---|---|---|---|---|---|---|---|---|---|---|---|
| 12 | 19/06/13 | 30/04/13 | 58 | f | KSA | NR | Al Hasa | Al Hasa | no | KSA | mild | recovered | no | --- | NR | no | yes | [21] |
| 12 | 19/06/13 | 01/05/13 | 48 | m | KSA | NR | Al Hasa | Al Hasa | no | KSA | severe | fatal | no | --- | NR | no | NR | [21] |
| 12 | 19/06/13 | 02/05/13 | 45 | m | KSA | NR | Al Hasa | Al Hasa | no | KSA | severe | in hospital | no | --- | NR | yes | no | [21] |
| 12 | 19/06/13 | 04/05/13 | 81 | m | KSA | NR | Al Hasa | Al Hasa | no | KSA | severe | in hospital | no | --- | NR | no | yes | [21] |
| 12 | 19/06/13 | 05/05/13 | 56 | m | KSA | NR | Al Hasa | Al Hasa | no | KSA | mild? | recovered | no | --- | NR | no | yes | [21] |
| 12 | 19/06/13 | 05/05/13 | 69 | f | KSA | NR | Al Hasa | Al Hasa | no | KSA | severe | fatal | no | --- | NR | no | NR | [21] |
| 12 | 19/06/13 | 06/05/13 | 48 | m | KSA | NR | Al Hasa | Al Hasa | no | KSA | severe | in hospital | no | --- | NR | no | yes | [21] |
| 12 | 19/06/13 | 08/05/13 | 42 | f | KSA | NR | Al Hasa | Al Hasa | no | KSA | mild? | recovered | no | --- | NR | yes | yes | [21] |
| 12 | 19/06/13 | 12/05/13 | 56 | m | KSA | NR | Al Hasa | Al Hasa | no | KSA | severe | fatal | no | --- | NR | no | yes | [21] |
| 12 | 04/06/13 | 27/05/13 | 83 | m | KSA | NR | Al Ahsa | Al Ahsa | no | KSA | severe | fatal | no | | NR | no | yes | [14] |
| 13 | 09/05/13 | 22/04/13 | 64 | m | France | NR | North France | Dubai | yes | France | severe | fatal | yes | Dubai 9-17 4/13 | NR | no | yes | [5, 14] |
| 13 | 12/05/13 | 08/05/13 | 51 | m | France | NR(assume french) | North France | North France | no | France | severe | in hospital | no | -- | no | no | yes | [5, 14] |
| 14 | 14/05/13 | 25/04/13 | 69 | f | KSA | KSA | eastern KSA | eastern KSA | no | KSA | severe | fatal | no | -- | NR | no | yes | [14] |
| 16 | 22/05/13 | NR | 34 | m | Tunisia | Tunisia | Monastir | Monastir | no | none | mild | recovered | No | -- | NR | no | NR | [14] |
| 16 | 22/05/13 | NR | 35 | f | Tunisia | Qatar | Monastir | Monastir | no | none | mild | recovered | Yes | NR | NR | no | NR | [14] |
| 17 | 22/05/13 | NR | NR | m | KSA | non-KSA | Al-Qaseem | Al-Qaseem | no | KSA | severe | fatal | NR | -- | NR | NR | NR | [14] |
| 18 | 28/05/13 | 12/05/13 | 56 | m | KSA | NR | eastern KSA | eastern KSA | no | KSA | severe | fatal | NR | -- | NR | no | yes | [14] |
| 18 | 28/05/13 | 17/05/13 | 85 | f | KSA | NR | eastern KSA | eastern KSA | no | KSA | severe | in hospital | NR | -- | NR | no | yes | [14] |
| 18 | 28/05/13 | 18/05/13 | 73 | m | KSA | NR | eastern KSA | eastern KSA | no | KSA | severe | fatal | NR | -- | NR | no | yes | [14] |
| 18 | 28/05/13 | 19/05/13 | 77 | m | KSA | NR | eastern KSA | eastern KSA | no | KSA | severe | fatal | NR | -- | NR | no | yes | [14] |
| 18 | 28/05/13 | 24/05/13 | 76 | f | KSA | NR | eastern KSA | eastern KSA | no | KSA | NR | recovered | NR | -- | NR | no | yes | [14] |
| 19 | 02/06/13 | NR | 2 | f | Italy | Italy | Florence | Italy | no | Italy | mild | NR | no | -- | no | no | NR | [14] |
| 19 | 02/06/13 | NR | 42 | f | Italy | Italy | Florence | Italy | no | Italy | mild | NR | no | -- | no | no | NR | [14] |
| 19 | 01/06/13 | NR | 45 | m | Italy | Italy | Florence | Jordan | yes | Italy | severe | in hospital | yes | Jordan 40 days | NR | no | NR | [14] |
| 20 | 05/06/13 | 29/05/13 | 14 | f | KSA | KSA | eastern KSA | KSA | no | KSA | NR | NR | no | -- | NR | no | yes | [14] |
| 21 | 12/06/2013 | NR | 21 | m | KSA | resident of KSA | Hafr Al Batin | eastern KSA | no | KSA | severe | fatal | no | -- | NR | no | NR | [14, 24] |
| 22 | 12/06/2013 | NR | 63 | f | KSA | KSA | KSA | eastern KSA | no | KSA | NR | in hospital | no | -- | NR | no | yes | [14, 24] |
| 23 | 14/06/2013 | NR | 65 | NR | KSA | KSA | Taif | Taif | no | KSA | severe | in hospital | no | -- | NR | no | yes | [14, 24] |
| 24 | 14/06/2013 | NR | 68 | f | KSA | KSA | Taif | Taif | no | KSA | severe | in hospital | no | -- | NR | no | yes | [14, 24] |
| 25 | 14/06/2013 | NR | 46 | m | KSA | NR | Wadi Al-Dawaser | Wadi Al-Dawaser | no | KSA | severe | fatal | no | -- | NR | no | NR | [14, 24] |
| 26 | 16/06/2013 | NR | 2 | NR | KSA | KSA | Jeddah | KSA | no | KSA | severe | in hospital | no | -- | NR | no | yes | [14, 24] |
| 27 | 16/06/2013 | NR | 42 | NR | KSA | KSA | eastern KSA | KSA | no | KSA | NR | in hospital | no | -- | NR | no | yes | [14, 24] |
| 28 | 16/06/2013 | NR | 63 | f | KSA | KSA | Riyadh | KSA | no | KSA | NR | in hospital | no | -- | NR | no | yes | [14, 24] |
| 29 | 22/06/2013 | NR | 43 | f | KSA | NR | Eastern Province | eastern KSA | no | KSA | NR | recovered | no | -- | NR | NR | no | [14, 24] |

| | | | | | | | | | | | | | | | | | | |
|---|---|---|---|---|---|---|---|---|---|---|---|---|---|---|---|---|---|---|
| 30 | 22/06/2013 | NR | 29 | f | KSA | NR | Taif | Taif | no | KSA | NR | recovered | no | -- | NR | NR | yes | 14, 24 |
| 30 | 22/06/2013 | NR | 39 | f | KSA | NR | Taif | Taif | no | KSA | asymptomatic | recovered | no | -- | NR | NR | yes | 14, 24 |
| 30 | 22/06/2013 | NR | 45 | f | KSA | NR | Taif | Taif | no | KSA | asymptomatic | recovered | no | -- | NR | NR | yes | 14, 24 |
| 8 | 23/06/2013 | NR | 41 | f | KSA | NR | Riyadh | Riyadh | no | KSA | NR | NR | no | -- | NR | NR | no | 14, 24 |
| 31 | 23/06/2013 | NR | 32 | m | KSA | NR | Eastern Province | eastern KSA | no | KSA | severe | in hospital | no | -- | NR | NR | no | 14, 24 |
| 8 | 26/06/2013 | NR | 7-15 | NR | KSA | NR | Riyadh | KSA | no | KSA | asymptomatic | recovered | no | -- | NR | no | no | 14, 24 |
| 8 | 26/06/2013 | NR | 7-15 | NR | KSA | NR | Riyadh | KSA | no | KSA | asymptomatic | recovered | no | -- | NR | no | no | 14, 24 |
| 8 | 26/06/2013 | NR | 7-15 | NR | KSA | NR | Riyadh | KSA | no | KSA | asymptomatic | recovered | no | -- | NR | no | no | 14, 24 |
| 8 | 26/06/2013 | NR | 7-15 | NR | KSA | NR | Riyadh | KSA | no | KSA | asymptomatic | recovered | no | -- | NR | no | no | 14, 24 |
| 32 | 26/06/2013 | NR | NR | f | KSA | NR | Eastern Province | KSA | no | KSA | asymptomatic | recovered | no | -- | NR | no | yes | 14, 24 |
| 33 | 26/06/2013 | NR | NR | f | KSA | NR | Al Hasa | KSA | no | KSA | asymptomatic | recovered | no | -- | NR | no | yes | 14, 24 |
| 34 | 26/06/2013 | NR | 50 | f | KSA | KSA | Eastern Province | KSA | no | KSA | NR | in hospital | no | -- | NR | no | no | 14, 24 |
| 35 | 05/07/2013 | NR | 69 | m | KSA | KSA | Riyadh | KSA | no | KSA | severe | in hospital | no | -- | NR | no | no | 14, 24 |
| 36 | 05/07/2013 | NR | 66 | m | KSA | KSA | Riyadh | KSA | no | KSA | severe | in hospital | no | -- | NR | no | no | 14, 24 |
| ? | 07/07/2013 | NR | 56 | f | KSA | NR | Eastern Province | KSA | no | KSA | NR | recovered | no | -- | NR | NR | yes | 14, 24 |
| 37 | 11/07/2013 | NR | 66 | m | KSA | KSA | Asir | KSA | no | KSA | severe | in hospital | no | -- | NR | no | no | 14, 24 |
| 38 | 13/07/2013 | 06/07/2013 | 82 | m | UAE | UAE | Silaa, Abu Dhabi | UAE | no | UAE | severe | in hospital | no | -- | NR | NR | no | 14, 24 |
| 37 | 18/07/2013 | NR | 26 | m | KSA | KSA | Asir | KSA | no | KSA | mild | recovered | no | -- | NR | no | no | 14, 24 |
| 37 | 18/07/2013 | NR | 42 | f | KSA | KSA | Asir | KSA | no | KSA | mild | recovered | no | -- | NR | no | yes | 14, 24 |
| 38 | 18/07/2013 | NR | 28 | m | UAE | UAE | Abu Dhabi | UAE | no | UAE | mild | recovered | NR | -- | NR | no | yes | 14 |
| 38 | 18/07/2013 | NR | 30 | f | UAE | UAE | Abu Dhabi | UAE | no | UAE | mild | recovered | NR | -- | NR | no | yes | 14 |
| 38 | 18/07/2013 | NR | 30 | f | UAE | UAE | Abu Dhabi | UAE | no | UAE | mild | recovered | NR | -- | NR | no | yes | 14 |
| 38 | 18/07/2013 | NR | 40 | f | UAE | UAE | Abu Dhabi | UAE | no | UAE | mild | recovered | NR | -- | NR | no | yes | 14 |
| 39 | 21/07/2013 | 15/07/2013 | 41 | m | KSA | KSA | Riyadh | KSA | no | KSA | severe | in hospital | no | -- | no | no | no | 14, 24 |
| 40 | 21/07/2013 | 11/07/2013 | 59 | f | KSA | KSA | Al Hasa | KSA | no | KSA | NR | NR | no | -- | no | no | no | 14, 24 |
| 41 | 29/07/2013 | 17/07/2013 | 83 | m | KSA | KSA | Asir | KSA | no | KSA | NR | in hospital | NR | -- | NR | no | no | 14, 24 |
| 42 | 01/08/2013 | 25/07/2013 | 67 | f | KSA | KSA | Riyadh | KSA | no | KSA | NR | in hospital | no | -- | no | no | no | 14, 24 |
| 37 | 01/08/2013 | NR | NR | f | KSA | KSA | Asir | KSA | no | KSA | mild | recovered | no | -- | no | no | yes | 14, 24 |
| 8 | 01/08/2013 | NR | NR | f | KSA | KSA | Riyadh | KSA | no | KSA | mild | recovered | no | -- | no | no | yes | 14, 24 |

| | | | | | | | | | | | | | | | | | | |
|---|---|---|---|---|---|---|---|---|---|---|---|---|---|---|---|---|---|---|
| 16* | 22/05/2013 | 01/05/2013 | 66 | m | Tunisia | Tunisia | Taif | Monastir | yes | tunisia | severe | fatal | yes | Qatar & KSA several weeks | NR | NR | no | 22 |
| 4* | 30/11/2012 | 30/03/2012 | 30 | m | Jordan | Jordan | Zarqa | Zarqa | no | Jordan | mild | recovered | no | -- | no | no | yes | 6 |
| 4* | 30/11/2012 | 02/04/2012 | 60 | m | Jordan | Jordan | Zarqa | Zarqa | no | Jordan | mild | recovered | no | -- | no | no | yes | 6 |
| 4* | 30/11/2012 | 11/04/2012 | 29 | m | Jordan | Jordan | Zarqa | Zarqa | no | Jordan | mild | recovered | no | -- | no | no | yes | 6 |
| 4* | 30/11/2012 | 12/04/2012 | 33 | m | Jordan | Jordan | Zarqa | Zarqa | no | Jordan | mild | recovered | no | -- | no | no | yes | 6 |
| 4* | 30/11/2012 | 13/04/2012 | 28 | m | Jordan | Jordan | Zarqa | Zarqa | no | Jordan | mild | recovered | no | -- | no | no | yes | 6 |
| 4* | 30/11/2012 | 14/04/2012 | 45 | m | Jordan | Jordan | Zarqa | Zarqa | no | Jordan | mild | recovered | no | -- | no | no | no | 6 |
| 4* | 30/11/2012 | 15/04/2012 | 46 | m | Jordan | Jordan | Zarqa | Zarqa | no | Jordan | mild | recovered | no | -- | no | no | yes | 6 |
| 4* | 30/11/2012 | 15/04/2012 | 25 | m | Jordan | Jordan | Zarqa | Zarqa | no | Jordan | mild | recovered | no | -- | no | no | yes | 6 |
| 4* | 30/11/2012 | 18/04/2012 | 53 | m | Jordan | Jordan | Zarqa | Zarqa | no | Jordan | mild | recovered | no | -- | no | no | yes | 6 |
| 4* | 30/11/2012 | 19/04/2012 | 28 | f | Jordan | Jordan | Zarqa | Zarqa | no | Jordan | mild | recovered | no | -- | no | no | yes | 6 |
| 4* | 30/11/2012 | 26/04/2012 | 60 | f | Jordan | Jordan | Zarqa | Zarqa | no | Jordan | mild | recovered | no | -- | no | no | no | 6 |
| 2* | NR | 03/11/2012 | 16 | m | KSA | KSA | Riyadh | Riyadh | no | KSA | mild | recovered | NR | NR | NR | NR | no | 8 |
| 10* | NR | NR | NR | m | KSA | KSA | Riyadh | Riyadh | no | KSA | NR | NR | NR | NR | NR | NR | no | 23 |
| 12* | 19/06/2013 | 11/04/2013 | 55 | m | KSA | NR | Al Hasa | Al Hasa | no | KSA | severe | fatal | no | --- | NR | NR | no | 21 |
| 12* | 19/06/2013 | 08/04/2013 | 56 | m | KSA | NR | Al Hasa | Al Hasa | no | KSA | severe | fatal | no | --- | NR | NR | no | 21 |
| 10* | 08/08/2013 | 13/02/2013 | 61 | m | KSA | KSA | Riyadh | KSA | no | KSA | severe | fatal | no | -- | yes | NR | no | 23 |

Notes: * probable case;  NR=not reported; m=male; f=female; KSA = Kingdom of Saudi Arabia; UAE = United Arab Emirates

## 2 Incubation period

To estimate the incubation period, detailed information on the exposure of secondary cases to the index case, in the absence of other potential exposures, was extracted from published description of traveler-related clusters reported from France [5], the United Kingdom [10], Italy [21] and Tunisia [22] (Table S2). Precise exposure and onset dates are not publically available for the Tunisia cluster but exposure windows for secondary cases have been reported. The advantage of restricting our analysis to secondary cases caused by recent travelers to affected countries is that the source of infection can be more reliably determined than for cases arising in affected countries such as Saudi Arabia.

In addition, the Al-Ahsa cluster in Saudi Arabia provides substantial information; we deliberately do not use those data in estimating the incubation period distribution here in order to generate a second independent estimate to be compared with existing estimates derived from analysis of that cluster [21].

We fitted the following probabilistic distributions to the data: Gamma, Log-Normal, Weibull. The distribution with the best Akaike Information Criterion is presented in the manuscript.

**Table S2. Estimated incubation period for individual MERS-CoV cases with available data, excluding Al Hasa cluster.**

| Country | First date of exposure | Last date of exposure | Symptom Onset | Incubation period (days; using symptom onset or exposure day 0) | | Source |
|---|---|---|---|---|---|---|
| | | | | Min | Max | |
| UK | 28/01/2013 | 31/01/2013 | 06/02/2013 | 6 | 9 | 10 |
| UK | 01/02/2013 | 04/02/2013 | 05/02/2013 | 1 | 4 | 10 |
| France | 26/04/2013 | 29/04/2013 | 08/05/2013 | 9 | 12 | 22 |
| Italy | 25/05/2013 | 26/05/2013 | 29/05/2013 | 3 | 4 | 21 |
| Italy | 27/05/2013 | 27/05/2013 | 31/05/2013 | 4 | 4 | 21 |
| Tunisia | NR | NR | NR | 3 | 3 | 22 |
| Tunisia | NR | NR | NR | 4 | 4 | 22 |

## 3 Delay between onsets in the first 1st and 2nd case in clusters

We consider clusters of probable and confirmed cases. Six clusters (clusters 2, 4, 7, 12, 13, 18 had more than 1 case with information on the date of symptom onset. In the Al Hasa cluster (cluster 12), the second case of the cluster (case B, [21]) is believed to have been infected in the community and is therefore excluded from this analysis. The delay between symptom onset in the first and the second case is given in Table S3.

We fitted the following probabilistic distributions to the data: Gamma, Log-Normal, Weibull. The distribution with the best Akaike Information Criterion is presented in the manuscript.

**Table S3. Delay between symptom onset in the first and the second case.**

| Cluster ID | Delay between symptom onset from 1st to 2nd case (days) |
|---|---|
| 2 | 19 |
| 4 | 9 |
| 7 | 12 |
| 12 | 3 |
| 13 | 16 |
| 18 | 5 |

A recent study estimated the mean serial interval to be 7.6 days[21]. However, this value was derived from a large case cluster in Al Hasa, Saudi Arabia, where infection control measures were intensified within two weeks of the detection of the outbreak, the effect of which would be to bias the observed serial intervals to be shorter than would be seen in the absence of control measures. The advantage of restricting our analysis to secondary cases caused by recent travellers to affected countries is that the source of infection can be more reliably determined than for cases arising in affected countries such as Saudi Arabia. It also provides a second independent estimate to be compared with the existing one[21].

# 4 Epidemic curve, exponential growth and reproduction number

For each cluster, we determine the earliest date of symptom onset in the cluster. When the date of symptom onset is unavailable, we subtract the median delay from 1st date of symptom onset to 1st date of reporting to estimate the 1st date of symptom onset. Since 19 of 22 clusters with missing onset date were reported after May 1st, we use the median delay onset-reporting for clusters reported after May 1st (=12 days). Table S4 shows the 1st date of symptom onset in each cluster.

**Table S4. Summary of cluster data.**

| Cluster ID | Cluster size | Date of first reporting | First onset onset date | Estimated first symptom onset date | Number of days since 1 Jan 2012 |
|---|---|---|---|---|---|
| 4 | 13 | 30/11/2012 | 21/03/2012 | 21/03/2012 | 80.00 |
| 1 | 1 | 20/09/2012 | 13/06/2012 | 13/06/2012 | 164.00 |
| 5 | 1 | 22/09/2012 | 03/09/2012 | 03/09/2012 | 246.00 |
| 6 | 1 | 01/11/2012 | 01/10/2012 | 01/10/2012 | 274.00 |
| 2 | 4 | 01/11/2012 | 05/10/2012 | 05/10/2012 | 278.00 |
| 3 | 1 | 04/11/2012 | 09/10/2012 | 09/10/2012 | 282.00 |
| 7 | 3 | 11/02/2013 | 24/01/2013 | 24/01/2013 | 389.00 |
| 8 | 7 | 21/02/2013 | NR | 09/02/2013 | 405.00 |
| 10 | 4 | 12/03/2013 | 13/02/2013 | 13/02/2013 | 409.00 |
| 9 | 1 | 07/03/2013 | NR | 23/02/2013 | 419.00 |
| 11 | 1 | 26/03/2013 | NR | 14/03/2013 | 438.00 |
| 12 | 26 | 04/06/2013 | 08/04/2013 | 08/04/2013 | 463.00 |
| 13 | 2 | 09/05/2013 | 22/04/2013 | 22/04/2013 | 477.00 |
| 14 | 1 | 14/05/2013 | 25/04/2013 | 25/04/2013 | 480.00 |
| 16 | 3 | 22/05/2013 | 01/05/2013 | 01/05/2013 | 486.00 |
| 17 | 1 | 22/05/2013 | NR | 10/05/2013 | 495.00 |
| 18 | 5 | 28/05/2013 | 12/05/2013 | 12/05/2013 | 497.00 |
| 19 | 3 | 01/06/2013 | NR | 20/05/2013 | 505.00 |
| 20 | 1 | 05/06/2013 | 29/05/2013 | 29/05/2013 | 514.00 |
| 21 | 1 | 12/06/2013 | NR | 31/05/2013 | 516.00 |
| 22 | 1 | 12/06/2013 | NR | 31/05/2013 | 516.00 |
| 23 | 1 | 14/06/2013 | NR | 02/06/2013 | 518.00 |
| 24 | 1 | 14/06/2013 | NR | 02/06/2013 | 518.00 |
| 25 | 1 | 14/06/2013 | NR | 02/06/2013 | 518.00 |
| 26 | 1 | 16/06/2013 | NR | 04/06/2013 | 520.00 |
| 27 | 1 | 16/06/2013 | NR | 04/06/2013 | 520.00 |
| 28 | 1 | 16/06/2013 | NR | 04/06/2013 | 520.00 |
| 29 | 1 | 22/06/2013 | NR | 10/06/2013 | 526.00 |
| 30 | 3 | 22/06/2013 | NR | 10/06/2013 | 526.00 |
| 31 | 1 | 23/06/2013 | NR | 11/06/2013 | 527.00 |
| 32 | 1 | 26/06/2013 | NR | 14/06/2013 | 530.00 |
| 33 | 1 | 26/06/2013 | NR | 14/06/2013 | 530.00 |
| 34 | 1 | 26/06/2013 | NR | 14/06/2013 | 530.00 |
| 35 | 1 | 05/07/2013 | NR | 23/06/2013 | 539.00 |
| 36 | 1 | 05/07/2013 | NR | 23/06/2013 | 539.00 |
| 37 | 4 | 11/07/2013 | NR | 29/06/2013 | 545.00 |
| 38 | 5 | 13/07/2013 | 06/07/2013 | 06/07/2013 | 552.00 |
| 40 | 1 | 21/07/2013 | 11/07/2013 | 11/07/2013 | 557.00 |
| 41 | 1 | 29/07/2013 | 17/07/2013 | 17/07/2013 | 563.00 |
| 42 | 1 | 01/08/2013 | 25/07/2013 | 25/07/2013 | 571.00 |

$^{+}$: The time of analysis is 8 August 2013, which corresponds to 585 days since Jan 1$^{st}$ 2012.

Because the discovery of an index case prompts enhanced surveillance in contacts of that case, the timing of first detected cases of clusters presented in Table S4 is likely more representative of the relative magnitude of the epidemic over time than the incidence of cases.

We fit simple non-homogeneous Poisson processes to these data to investigate how the risk of cluster occurrence/detection $\lambda(t)$ changed with time t. The risk $\lambda(t)$ quantified here is a composite of the rate at which clusters happen and the risk they are effectively detected. Denote $t_i$ the first date of symptom onset for the *i*-th cluster (*i*=1,…,*n*) with times defined since 1st January 2012 (time $t_0$) and *T* the final date considered in these analyses (11 June 2013). The likelihood of the sequence of first detections of clusters is, for a non-homogeneous Poisson process:

$$L = \exp\left\{-\int_{t_n}^{T} \lambda(u)\,du\right\} \prod_{i=1}^{n} \lambda(t_i) \exp\left\{-\int_{t_{i-1}}^{t_i} \lambda(u)\,du\right\}$$

We consider different models that are fitted to the data by maximum likelihood with 95% confidence intervals derived using a profile likelihood. The Akaike Information Criterion (AIC) is used for model comparison.

In model *M₀*, we assume the risk of cluster occurrence/detection is invariant with time

$$\lambda^0(t) = \alpha_0$$

In model $M_1$, we assume the risk of cluster occurrence/detection is growing exponentially with time

$$\lambda^1(t) = \alpha_1 \exp(r_1 t)$$

In model $M_2$, the risk of cluster occurrence/detection is a step function with a sudden change on day *D*

$$\lambda^2(t) = \begin{cases} \alpha_2 & \text{if } t < D \\ \alpha_2\beta_2 & \text{otherwise} \end{cases}$$

Table S5 gives the estimates. On the basis of AIC, these data provide strong support for Models 1 and 2 over Model 0. Model 2 appears to be marginally better than model 1 although a sudden 10 fold rise in reporting at the end of April seems to be an extreme scenario.

**Table S5. Fit of simple models for the risk of cluster occurrence/detection over time.**

| Model | Parameter estimates | Maximum Log likelihood | AIC |
|---|---|---|---|
| Model 0 | $\alpha_0$=0.068 [0.049, 0.092] | -147.31 | 296.62 |
| Model 1 | $\alpha_1$=0.0034 [0.0009, 0.0109]<br>$r_1$=0.0077 [0.0052, 0.0106] | -124.53 | 253.06 |
| Model 2 | $\alpha_2$=0.0254 [0.0147, 0.0421]<br>$\beta_2$=10.3 [5.4, 21.0]<br>*D*=22 April 2013 [26 March 2013, 25 April 2013] | -121.99 | 249.98 |

Denoting *r* the exponential growth rate, the doubling time is $D = \ln(2)/r$ .

Furthermore, assume that the epidemic is driven by human-to-human transmission and that the generation time has a Gamma distribution with mean $\mu$ and standard deviation $\sigma$ . There is the following relationship between the reproduction number *R*, the exponential growth rate *r* and parameters of the Gamma distribution ( $a = \mu^2/\sigma^2$ and $b = \mu/\sigma^2$ ):

$$R = (1 + r/b)^a$$

As a sensitivity analysis, we estimate the reproduction number from the growth in incident cases. We obtain estimates equal to 1.08 (95% CI: 1.06, 1.09) for $T_G$=12 days and 1.04 (1.03, 1.05) $T_G$=7 days, *i.e.* similar to those presented in the manuscript, which are based on incident clusters.

## 5 Reproduction number averaged across all cases in the cluster ($R_{cluster}$)

Estimates of $R_{cluster}$ are derived from the distribution of cluster sizes (Table S4) using on standard branching process theory. We denote the length of a chain of transmission by *L*. Following Lloyd-Smith el al [25], the offspring distribution (*i.e.* number of persons infected by a case) is modelled with a Negative Binomial distribution with mean *R* and overdispersion parameter *k* (parameter *k* characterizes case-to-case variation in infectiousness). The probability that a chain is of length *L* is given by [26]:

$$g(L \mid R, k) = \frac{\Gamma((k+1)L-1)}{\Gamma(kL+1)\Gamma(L)} \frac{k^{kL+1} R^{L-1}}{(R+k)^{(k+1)L-1}}$$

We assume *k*=1 and calculate maximum likelihood estimates of $R_{cluster}$. Ninety five percent profile likelihood confidence intervals are also calculated.

# 6 Reproduction number of the index case in clusters (*$R_{index}$*)

We derive the reproduction number of the index case (i.e. the case with the earliest date of symptom onset) in each cluster, based on an estimate of the number of cases that were in the second generation of the cluster.

We restrict the analysis to clusters with complete information on symptom onset dates and with a first symptom onset date before 1 June 2013. This is a time period when 93% (13 of 14) of delays from onset to reporting of cluster were ≥16 days. In single case clusters, we assume $R_{index}$=0. In clusters with more than 2 cases and for whom dates of symptom onset are available, we use standard statistical methods (see Wallinga and Teunis, [27]) to probabilistically reconstruct the transmission tree and derive the reproduction number of the index case for $T_g$=12 days and $T_g$=7 days. Among the 12 clusters with complete onset information and a first date of onset before 1 June 2013, 6 are single case clusters and 6 have at least 1 secondary case. Table S6 presents estimates of $R_{index}$ for the 6 clusters with at least 1 secondary case with complete onset information.

In a sensitivity analysis, we provide a conservative estimate of $R_{index}$ based on all clusters including those with incomplete onset information, with estimated symptom onset before 1 June 2013. In single case clusters, we know that $R_{index}$=0 even if the onset date is missing. But estimation of $R_{index}$ for larger clusters requires the timing of symptoms onset [27]. We cannot simply exclude these clusters from analysis as this would bias estimates downward. In 2 clusters (Tunisian cluster 16 and Italian cluster 19), dates of symptoms onset are unavailable but there is good epidemiological evidence that the index case infected the 2 secondary cases [14]. We therefore assume $R_{index}$=2 in these 2 clusters. For 2 other clusters with missing data on onset dates (cluster 8 and cluster 10), we are conservative and assume that $R_{index}$ was equal to 1. There are 5 single case clusters with missing onset dates for which we assume $R_{index}$=0. We obtain $R_{index}$=1.00 (95% CI: 0.86, 1.14) for $T_G$=12 days and $R_{index}$=0.76 (95% CI: 0.66, 0.90) for $T_G$=7 days. Clusters 8 and 10 had size 7 and 4, respectively. Assuming that Rindex was equal to 2 in these clusters, the estimate would become 1.10 (95% CI: 0.95, 1.24) $T_G$=12 days and 0.86 (95% CI: 0.76, 1.00) for $T_G$=7 days.

Table S7 summarizes estimates obtained in the different analyses.

**Table S6. Estimates of the reproduction number of the index case in clusters with more than 1 case, with complete onset information and with first onset before 1 June 2013. The mean generation time is assumed to be $T_G$=12 days and $T_G$=7 days.**

| Cluster ID | Location | Year | Total size (confirmed and probable) | Times of symptom onset in sec. cases (time 0=onset in index case) | $R_{index}$ ($T_G$=12d) | $R_{index}$ ($T_G$=7d) |
|---|---|---|---|---|---|---|
| **2** | KSA | 2012 | 4 | 19-29-30 | 1.04[0.1,2] | 1.00 [1,1] |
| **4** | Jordan (Zarqa) | 2012 | 13 | 9-12-12-21-22-23-24-25-25-28-29-36 | 2.81 [1,4] | 1.43 [1,3] |
| **5** | UK | 2012 | 1 | | 0 | 0 |
| **6** | Germany | 2012 | 1 | | 0 | 0 |
| **7** | UK | 2013 | 3 | 12-13 | 1.97 [1,2] | 1.53 [1,2] |
| **12** | KSA (Al Hasa) | 2013 | 26 | (1)-3-6-7-8-10-11-11-11-12-14-15-17-19-20-22-23-24-26-27-27-28-30-34-49* | 4.45 [2,7]$ | 2.89 [1,5]$ |
| **13** | France | 2013 | 2 | 16 | 1 | 1 |
| **14** | KSA | 2013 | 1 | | 0 | 0 |
| **18** | KSA | 2013 | 5 | 5-6-7-12 | 3.14 [2,4] | 2.59 [1,4] |

*: The times of symptom onset are used to estimate the reproduction number of the index case with the Wallinga and Teunis approach [27].

$: if case B (i.e the one with onset on day 1; [21]) is removed from the analysis R=4.87 [2,8] for $T_G$=12 day and R=2.75 [1,5] for $T_G$=7 days.

**Table S7. Estimates of the reproduction number in index cases of clusters with estimated onset date before 1 June 2013. Estimates are for a mean generation time $T_G$=12 days and $T_G$=7 days.**

| Analysis | $R_{index}$ ($T_G$=12 days) | $R_{index}$ ($T_G$=7 days) |
|---|---|---|
| **Baseline**<br>Restricted to clusters with complete onset information | 1.25 [1.00,1.50] | 0.83 [0.67,1.08] |
| **Sensitivity analysis 1**<br>All clusters<br>Assuming $R_{index}$=2 in Tunisian and Italian clusters<br>Assuming $R_{index}$=1 in 2 other with more than 1 case but missing onset data | 1.00 [0.86,1.14] | 0.76 [0.66,0.90] |
| **Sensitivity analysis 2**<br>All clusters<br>Assuming $R_{index}$=2 in Tunisian and Italian clusters<br>Assuming $R_{index}$=2 in 2 other with more than 1 case but missing onset data | 1.10 [0.95,1.24] | 0.86 [0.76,1.00] |

# 7 Using cases among returning non-resident travellers to infer comparable domestic case numbers

By 8 August 2013, four cases have been identified (in France, Italy, Tunisia and the UK) among returning non-resident travellers to Middle Eastern countries. Although this number is small, it is possible to use it to estimate the magnitude of comparable cases within Middle Eastern countries that may have occurred testing the assumption that case detection is

much more sensitive for returning non-resident travellers than it is for residents of the affected region.  These four cases were recorded as having visited the Jordan, Kingdom of Saudi Arabia, Qatar and United Arab Emirates (see Figure S1).

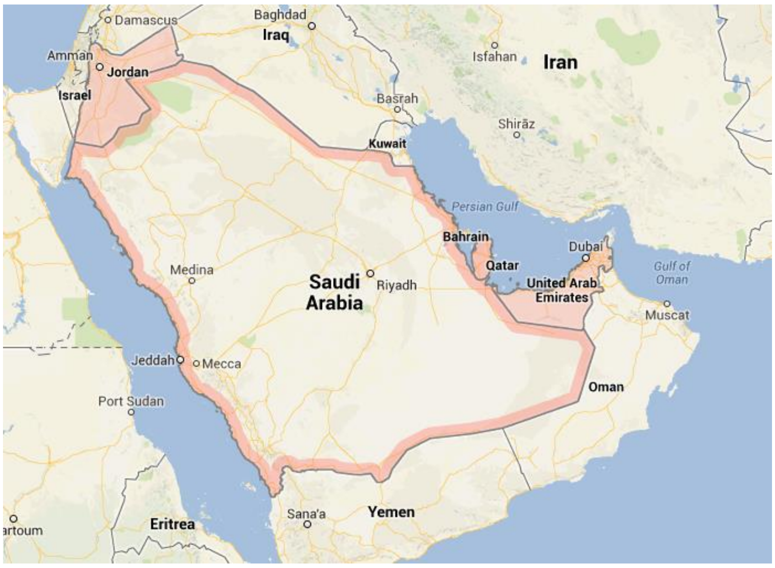


**Figure S1**. This map highlights the four countries the Jordan, Kingdom of Saudi Arabia, Qatar and United Arab Emirates from which returning non-resident travellers have subsequently been diagnosed with MERS-CoV.

Estimated 2013 population sizes were obtained for Jordan, Kingdom of Saudi Arabia, Qatar and United Arab Emirates (Table S8).  These were used to calculate domestic person-days at risk (per annum) by multiplying these numbers by 365.

The person-days at risk for returning non-resident travellers were estimated by multiplying the number of inbound overnight tourist arrivals per annum (in 2011, excluding those from the Middle East) by the average length of stay (estimated to be 4.0 days for inbound tourists visiting the Kingdom of Saudi Arabia in 2011 – World Tourism Organisation, 2012).  The numbers of inbound overnight tourist arrivals in 2011 were as given in Table S8.

We restricted our analysis to travellers from outside the Middle East to ensure we avoided confusing travel-related infections with locally transmitted infections. The data from World Tourism Organization did not exclude those who travelled on pilgrimages.

**Table S8. The population sizes and numbers of inbound tourist arrivals for Jordan, Kingdom of Saudi Arabia, Qatar and United Arab Emirates, excluding tourists from within the Middle East.**

| Country | Population size° | Number of inbound tourist arrivals* EXCLUDING those from the Middle East |
|---|---|---|
| Jordan | 6,482,081 | 2,066,000 |
| KSA (Saudi Arabia) | 26,939,583 | 6,399,000 |
| Qatar | 2,042,444 | 1,699,000 |
| United Arab Emirates | 5,473,972 | 5,786,315 |

°Source: Central Intelligence Agency World Factbook. https://www.cia.gov/library/publications/the-world-factbook/rankorder/2119rank.html

*Except for the United Arab Emirates (UAE), the numbers of inbound tourist arrivals for 2011 were obtained from the World Tourism Organization (2012), Compendium of Tourism Statistics dataset [Electronic], UNWTO, Madrid, data updated in December 2012. For UAE, the number of inbound tourist arrivals was estimated from the Abu Dhabi and Dubai Total Hotel Establishment Guests by Nationality, excluding those from Arab countries obtained from the World Tourism Organization (2012), Compendium of Tourism Statistics dataset [Electronic], UNWTO, Madrid, data updated in December 2012.

The number of comparable domestic cases is then estimated by multiplying 4 (the number of cases identified among returning non-resident travellers to Middle Eastern countries) by the ratio of domestic person-days at risk to returning non-resident traveller person-days at risk. In this case:

$$4 \times 14{,}942{,}399{,}200 / 63{,}801{,}260 = 937 \text{ domestic cases.}$$

However, this depends crucially on the assumed average length of stay. It is worth noting that the average was estimated to be 2.0 days for inbound tourists visiting Jordan in 2011 (World Tourism Organisation, 2012). However, as Saudi Arabia is has so many more inbound tourists than Jordan, the Saudi Arabia mean (4.0 days) is more indicative of the region as a whole. Table S9 shows the dependence of estimate on the assumed average length of stay as well as the upper and lower confidence limits based on the Poisson-likelihood-based 95% confidence bounds for the underlying rate of cases among returning non-resident travellers. These bounds do not incorporate uncertainty in either the country population sizes nor in the numbers of inbound tourists.

**Table S9. Sensitivity to the assumed average length of stay of estimated number of domestic cases in Jordan, Kingdom of Saudi Arabia, Qatar and United Arab Emirates combined, based on cases among returning non-resident travellers.**

| Average length of stay (in days) | Estimate | Lower Bound | Upper Bound |
|---|---|---|---|
| 1 | 3,747 | 1,163 | 8,705 |
| 2 | 1,874 | 582 | 4,353 |
| 3 | 1,249 | 388 | 2,902 |
| 4 | 937 | 291 | 2,176 |
| 5 | 749 | 233 | 1,741 |
| 6 | 625 | 194 | 1,451 |
| 7 | 535 | 166 | 1,244 |
| 8 | 468 | 145 | 1,088 |
| 9 | 416 | 129 | 967 |
| 10 | 375 | 116 | 871 |
| 11 | 341 | 106 | 791 |
| 12 | 312 | 97 | 725 |
| 13 | 288 | 89 | 670 |
| 14 | 268 | 83 | 622 |

These could be subdivided into the separate countries based on the proportion of inbound tourist arrivals to the four countries in total that were to a particular country (Table S10).

**Table S10. Country-specific estimates of domestic cases based on cases among returning non-resident travellers.**

| Average length of stay (in days) | Jordan | Saudi Arabia | Qatar | United Arab Emirates | Total |
|---|---|---|---|---|---|
| 2 | 297<br><br>(92 – 689) | 1233<br><br>(383 – 2864) | 93<br><br>(29 – 217) | 251<br><br>(78 -582) | 1,874<br><br>(582 – 4353) |
| 4 | 148<br><br>(46 – 345) | 616<br><br>(191 – 1,432) | 47<br><br>(15 – 109) | 125<br><br>(39 – 291) | 937<br><br>(291 – 2,176) |
| 7 | 85<br><br>(26 – 197) | 352<br><br>(109 – 818) | 27<br><br>(8 – 62) | 72<br><br>(22 – 166) | 535<br><br>(166 – 1244) |
| 10 | 59<br><br>(18 – 138) | 247<br><br>(77 – 573) | 19<br><br>(6 – 43) | 50<br><br>(16 – 116) | 375<br><br>(116 – 871) |

Although this is a simple approximation to a complex system, it yields useful estimates for cases in the affected Middle Eastern countries comparable to the four diagnosed among returning non-resident travellers. In particular, these estimates do not include mild cases that would not have warranted testing had they occurred in returning non-resident travellers. This does not allow for the fact that some comparable cases might well have occurred among returning non-resident travellers that returned to countries that do not test for MERS-CoV regardless of health status and travel history. It implicitly assumes that

the per-day risk of infection of domestic citizens and returning non-resident travelers are the same. Of course, returning non-resident travelers might well have a higher or lower per-day risk than the domestic population, depending on the route(s) of transmission and any within-country regional variation in risk.

As a sensitivity analysis, Table S11 gives the country-specific estimates for Kingdom of Saudi Arabia, Qatar and United Arab Emirates, based on 3 cases identified among returning non-resident travellers to Middle Eastern countries (i.e. excluding the most recent such case identified in a returning non-resident travellers who visited Jordan).

**Table S11. Country-specific estimates of domestic cases based on 3 cases identified among returning non-resident travellers to Middle Eastern countries (i.e. excluding the most recent such case identified in a returning non-resident traveller who visited Jordan).**

| Average length of stay (in days) | KSA (Saudi Arabia) | Qatar | United Arab Emirates | Total |
|---|---|---|---|---|
| 2 | 1062<br>(440 – 3,290) | 81<br>(33 – 249) | 216<br>(89 – 669) | 1,359<br>(562 – 4,208) |
| 4 | 531<br>(220 – 1,645) | 40<br>(17 – 125) | 108<br>(45 – 334) | 679<br>(281 – 2,104) |
| 7 | 304<br>(126 – 940) | 23<br>(10 – 71) | 62<br>(26 – 191) | 388<br>(161 – 1,202) |
| 10 | 212<br>(88 – 658) | 16<br>(7 – 50) | 43<br>(18 – 134) | 272<br>(112 – 842) |

# 8 Balance between animal-to-human and human-to-human transmission

We illustrate the balance between animal-to-human and human-to-human transmission under different assumptions about the human-to-human reproduction number using a parsimonious mathematical model, with discrete generations. We fit the model jointly to two epidemiological data, the timing of clusters (that inform growth rates) and the exported cases (that inform the cumulated number of cases), for different values of $R_0$.

We model infection in an animal host that is assumed to seed infections into humans. Animal hosts can be infected by other animals, plus we include a low constant background risk of infection to prevent epidemic extinction in the animal host. The number $I_A(g)$ of infected animals at generation *g* is the sum of the number infected by the background risk, $I_{B\to A}(g)$, assumed to be Poisson distributed with mean $\lambda_{B\to A}$ and $I_{A\to A}(g)$, the number infected by other animals. Assuming a Negative Binomial distribution of the offspring distribution in animals with reproduction number $R_A$ and overdispersion parameter $k_A$, we have

$$I_{A\to A}(g) \sim NegBin\{I_A(g-1)r_A, 1-p_A\}$$

with $p_A = 1/(1+k_A/R_A)$ and $r_A = R_A(1-p_A)/p_A$.

At generation *g*, the number of human infections caused by animals is Poisson distributed $I_{A\to H}(g) \sim Pois(\gamma I_A(g))$. Assuming a Negative Binomial distribution of the offspring distribution in humans with reproduction number *R* and over-dispersion parameter *k*, the number of events of human-to-human transmission at generation *g* is

$$I_{H\to H}(g) \sim NegBin\{I_H(g-1)r, 1-p\}$$

with $p = 1/(1+k/R)$ and $r = R(1-p)/p$.

Given the (unobserved) number of human cases $I_H(g)$ at generation *g*, the observed number of cases $O_H(g)$ at that generation has a Binomial distribution

$$O_H(g) \mid I_H(g) \sim Bin(I_H(g), r)$$

where $\rho$ is the detection rate.

Last, given the total number of cases $\sum_g I_H(g)$, the observed number of exported case $n_E$=4 is Poisson distributed with mean $\sum_g I_H(g) / 234.2$ (see section 6 describing the analysis of exported cases).

For $\lambda_{B\to A} = 1$ per week, $k_I = 1$ and $k = 1$, we use Particle Markov chain Monte Carlo [28] to fit the model jointly to the data for different values of *R* and for generation times of 12 and 7 days. We use 3,000 particles and 30,000 MCMC iterations per run, with a burn in of 500 and derive the posterior distribution of parameters as well as the reconstructed trajectories of human infections due to the animal host and those due to human-to-human transmissions. We use the Deviance Information Criterion (DIC) for model comparison[29]. Smaller values of the DIC indicate a better fit. A difference of 5 DIC units is considered to be substantial.

Figures S2-S5 present for the different models the DICs, the estimated cumulated number of cases, proportion of human-to-human transmission so far and the probability that current chains of transmission will be sustained for a finite period (1 year), respectively.

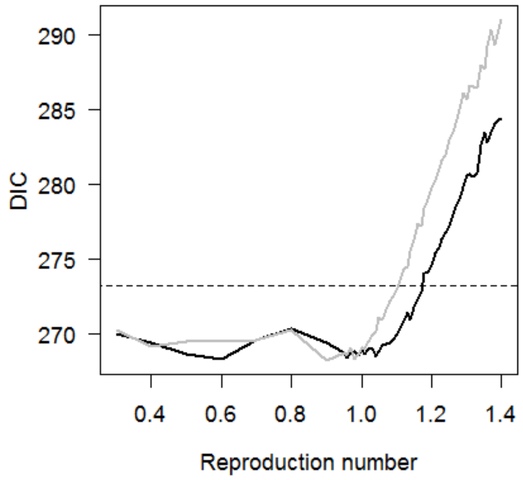


**Figure S2. DIC as a function of *R* for $T_g$=12 and 7 days.** The dotted line indicates the threshold for a substantial difference from the best fitting model (5 DIC units).

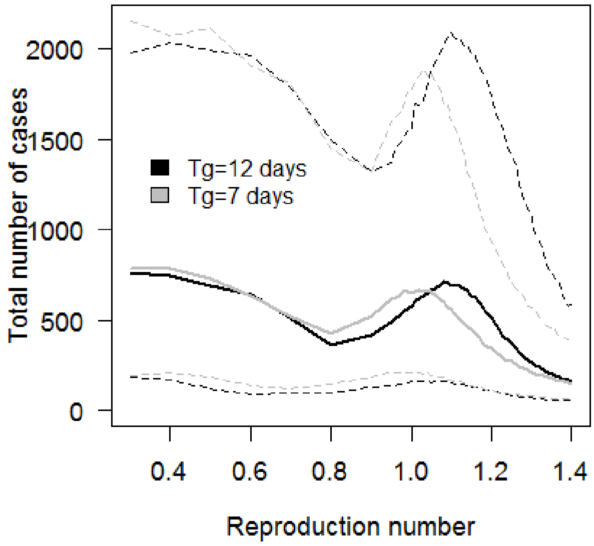


**Figure S3: Total number of cases as a function of *R* for $T_g$=12 and 7 days (solid line: median; dashed line: 95% CI).**

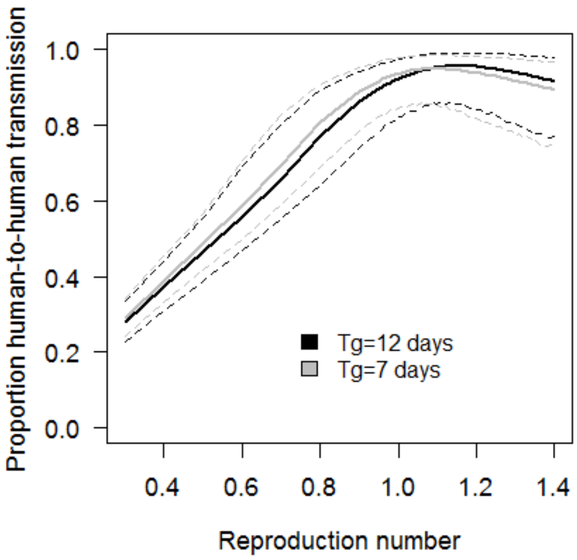


**Figure S4. Proportion of human-to-human transmission in the epidemic so far as a function of *R* for $T_g$=12 and 7 days (solid line: median; dashed line: 95% CI).**

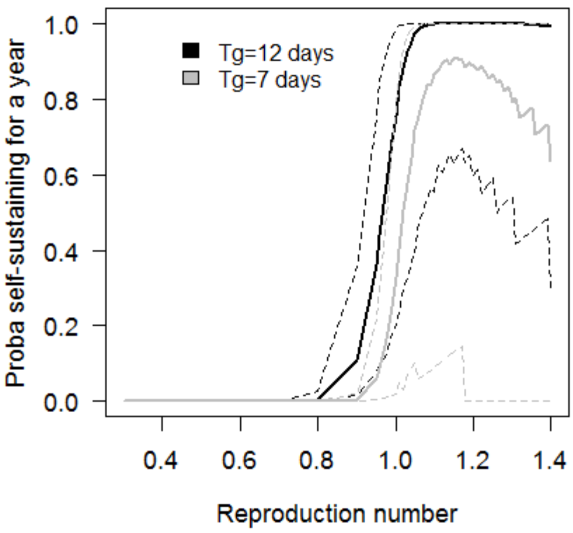


**Figure S5. Probability that current chains of transmission will be sustained for 1 year into the future as a function of *R* for $T_g$=12 and 7 days (solid line: median; dashed line: 95% CI).**

# 9 Summary of genetic analysis of MERS-CoV

## 9.1 The data

There are currently nine publicly available full-length MERC-CoV sequences. We also include in our analysis a sequence generated from a viral isolate of a patient diagnosed in France, who had previously travelled and presumably been infected in Dubai.

**Table S12. Isolates for which genetic data were available**

| Accession number | Name | Date of isolation | Cluster ID |
|---|---|---|---|
| KC776174 | Jordan-N3 | 2012-04-14 | 4 |
| JX869059 | KSA/EMC | 2012-06-13 | 1 |
| KC667074/ KC16505* | Qatar/England 1 | 2012-09-12 | 5 |
| PHE website** | England2 | 2013-02-10 | 7 |
| KF192507 | AbuDhabi/ Munich | 2013-03-22 | 11 |
| KF186564 | KSA/Al Hasa 1 | 2013-05-09 | 12 |
| KF186565 | KSA/Al Hasa 2 | 2013-04-21 | 12 |
| KF186566 | KSA/Al Hasa 3 | 2013-04-22 | 12 |
| KF186567 | KSA/Al Hasa 4 | 2013-05-01 | 12 |
| KF745068 | France-UAE/1627/2013 | 2013-05-07 | 13 |

*The first English isolate was sequenced twice, by Public Health England (formerly HPA) with accession number KC16505 and once at the Wellcome Trust Sanger institute (KC667074), which is the sequence included here[30].
**http://www.hpa.org.uk/ webc/HPAwebFile/HPAwebC/1317138176202
***The sequence of the isolate Hu/France-UAE/1627/2013 (Dubai/France 1) patient was generated at the Pasteur Institute in Paris using Sanger sequencing, essentially as described[31] and will be posted on Genbank as this paper is published.

In aligning these sequences, UTRs were ignored as they could be problematic to assemble, and inter-genic spacers as they were conserved. Coding regions were aligned using Muscle to produce an in-frame alignment.

## 9.2 Choosing the substitution model for phylogenetic analysis

Different substitution models were tested using jModelTest v2.1.3, and we report only values for common models (TN93, GTR and HKY).

**Table S13. Comparison of alternate substitution models.**

| Model | -LL | N Params | BIC |
|---|---|---|---|
| TN93 | 41936 | 17 | 84047 |
| TN93+Γ | 41932 | 18 | 84049 |
| TN93+I | 41936 | 18 | 84057 |
| TN93+I+Γ | 41932 | 19 | 84059 |
| HKY | 41948 | 16 | 84062 |
| GTR | 41928 | 20 | 84062 |
| HKY+Γ | 41945 | 17 | 84064 |
| GTR+Γ | 41924 | 21 | 84064 |
| HKY+I | 41948 | 17 | 84072 |
| GTR+I | 41928 | 21 | 84072 |
| HKY+I+Γ | 41945 | 18 | 84074 |
| GTR+I+Γ | 41924 | 22 | 84074 |

Based on this analysis, we use the TN93 substitution model for our main analysis, and perform sensitivity analysis to this choice below, with the HKY and GTR models, and also the SRD06 model, which allows for different substitution rates at third base positions in codons.

## 9.3 Maximum likelihood phylogeny

The maximum likelihood phylogeny was estimated using PhyML with the TN93 model, with 1,000 bootstrap iterations. Within this tree, we highlight in red a clade from within which all recent samples were drawn. Given the topology of the tree, we hypothesise that this clade is more likely to include samples from an outbreak of sustained human-to-human transmission, and that the other two sequences not in this clade were independent zoonotic introductions. This hypothesis could only be proved with identification and extensive sampling of the animal reservoir for this new virus.

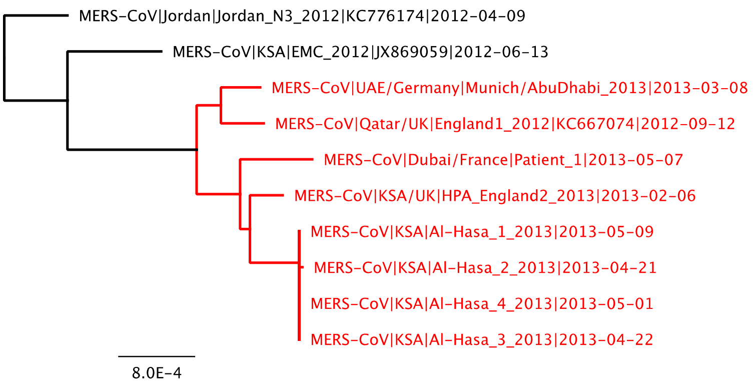


**Figure S6. Maximum likelihood phylogeny** estimated using PhyML with the TN93 model**.**

## 9.4 Evidence for a molecular clock

For all further modelling and hypothesis testing, we include only one sequence from the Al-Hasa outbreak (Al-Hasa 1, the most recent) as these are not independent from each other. We find very strong evidence of clock-like evolution, which supports the use of a molecular clock analysis.

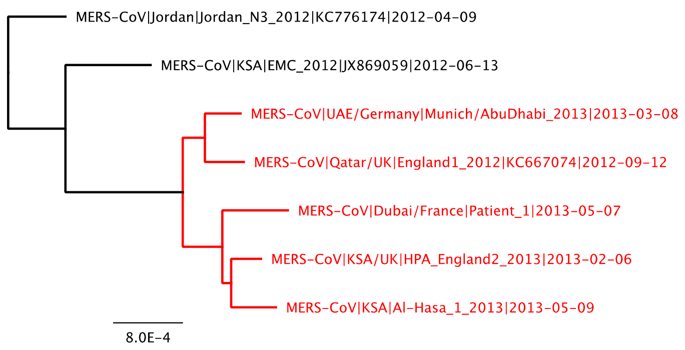


**Figure S7. Tree with highest correlation between root-to-tip distance and calendar time.**

The tree shown in Figure S7 gives the highest possible correlation between root-to-tip distance and calendar time, as illustrated by the regression shown in Figure S8.

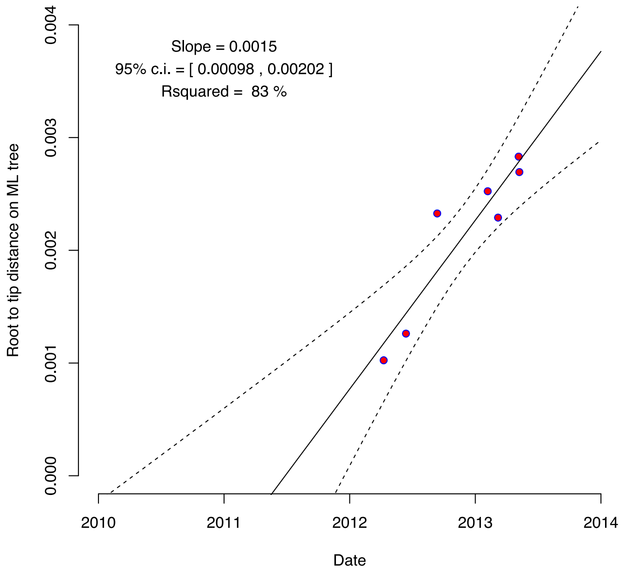


**Figure S8. Regression of root-to-tip distance against calendar time for tree shown in Figure S7.**

For this tree, the correlation between calendar time and root-to-tip distance tree is high ($R^2$=83%) with an estimated molecular clock rate of 1.50 $\times 10^{-3}$ substitutions per site per year (95% confidence interval 0.98 to 2.02 $\times 10^{-3}$), and an estimate of the TMRCA of mid 2011.

However, we note that the choice of root for this tree need not be based on the highest correlation of root-to-tip distance and time. There is alternative rooting of the tree that fits the data well, and gives a lower estimate of the clock rate. The alternative choice of root for the tree is shown in Figure S9.

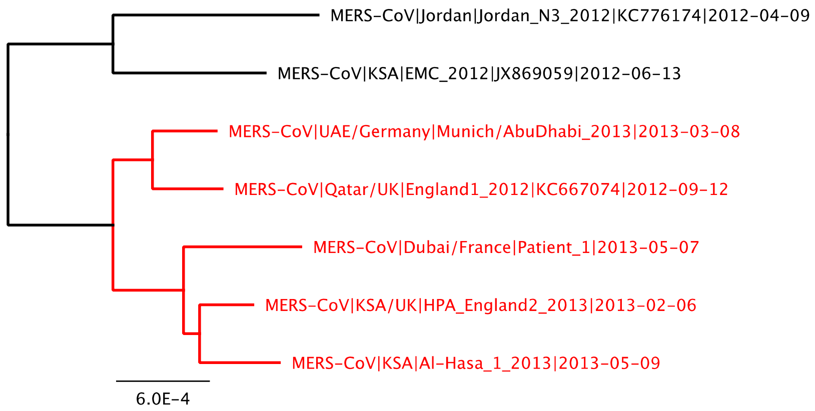


**Figure S9. Maximum likelihood phylogeny with alternative root.**

This choice minimizes the sum of square residuals in the regression between root-to-tip distance in the tree and calendar time, as illustrated by the regression shown in Figure S10.

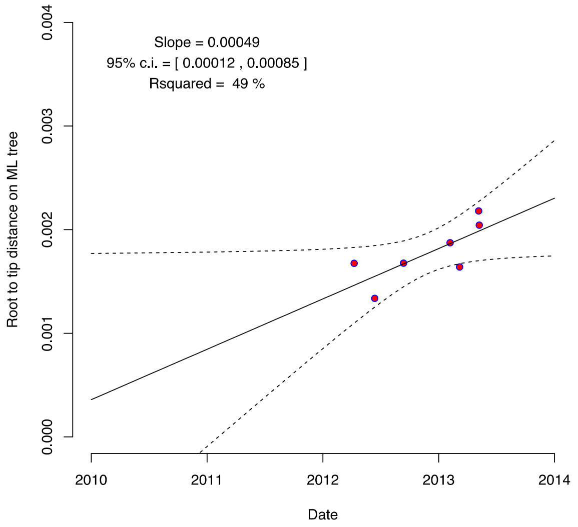


**Figure S10 Regression of root-to-tip distance against calendar time for tree shown in Figure S9.**

For this choice of tree, the correlation between the root-to-tip distance in this tree is moderate ($R^2$=49%) and suggests instead an estimate of the molecular clock rate of 0.49 × $10^{-3}$ substitutions per site per year (95% confidence interval 0.12 to 0.85 × $10^{-3}$).

The first choice of root for the tree seems more consistent with epidemiological evidence, given the first human case was reported in early 2012. Both choices of clock rate are broadly consistent with estimates from other coronaviruses and in particular the 2003 SARS epidemic. At this stage, it is not possible to definitively root the tree. This would likely require other early samples, or identification and sampling of the source animal reservoir.

Molecular evolutionary rates have been estimated for a few different coronaviruses for complete genomes or subgenomic regions.  Probably the most pertinent estimate would be

for the other known beta coronavirus in humans, SARS-CoV. But here there is also a disparity in rate estimates from $4{\times}10^{-4}$ for the large and relatively conserved ORF1ab open reading frame[32] to $1.5 \times 10^{-3}$ based on complete genomes [33].

Based on epidemiological plausibility, we focus on the estimate of $1.5 \times 10^{-3}$ per site per year in our main analysis, and perform an extensive sensitivity analysis to this choice of clock.

## 9.5 Bayesian coalescent inference of population dynamics

We focus on the recent clade of the virus, and so work with an alignment of five virus sequences, highlighted in red in the trees above. The rooting of this clade is unambiguous when considered in the light of the earlier sequences, and so we enforce this rooting in the analysis of the highlighted in-group.

### 9.5.1 Methods

We use BEAST [34] to estimate the dated phylogeny and the changing population size, using exponential population change for the coalescent model. We use a fixed value of the strict molecular clock. As a sensitivity analysis, we vary it between $5.0 \times 10^{-4}$ and $2.5 \times 10^{-3}$ in increments of $10^{-4}$ substitutions per site per year. We use the TN93 substitution model, but consider other models in a sensitivity analysis. We use a coalescent model with an exponentially changing population size. We assume a very broad and symmetric prior for the exponential rate of change, $r$ (prior is normal with mean 0 and standard deviation 100 per year). We assign all other priors to the default values used by BEAST (v1.7.4). Using a fixed clock, convergence of the MCMC chains was excellent. We emphasise that it is not a prior assumption that the infected population is growing, since the population size parameter and the rate of exponential change parameters are independent.

### 9.5.2 Dated phylogeny

Figure S11 shows the estimates dated phylogeny drawn from the posterior distribution (maximum clade credibility), based on the substitution rate of $1.5 \times 10^{-3}$

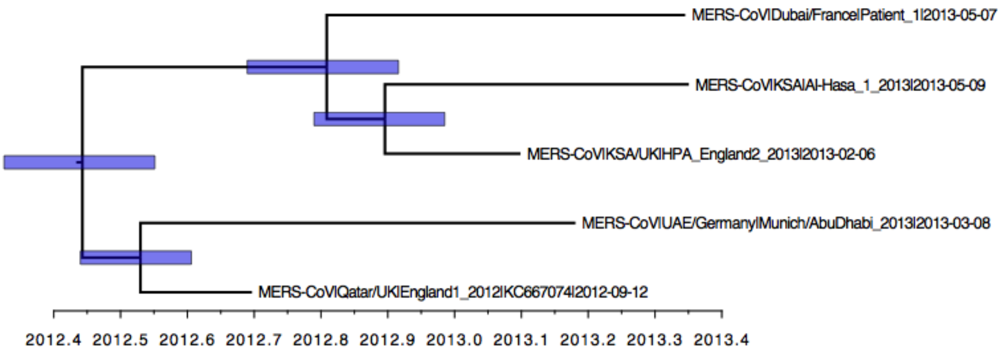


**Figure S11. Time resolved phylogeny of the recent clade of 5 samples.**

### 9.5.3 Population dynamics

Posterior estimates of the date of the most recent common ancestor (MRCA) and of the exponential growth rate are output from BEAST. The posterior distribution of the basic reproduction number $R_0$ are obtained by noting that in a growing epidemic, $R_0$ is related to

the growth rate $r$ and the generation time distribution by $R_0 = M(-r)^{-1}$, where $M$ is the moment generation function of the generation time distribution [35]. We assume a Gamma distributed generation time with mean $Tg$ and shape coefficient $\alpha$, so that $R_0(r)=(1+rT_g/\alpha)^{\alpha}$ . In our baseline scenarios we assume that $T_g$ is 12 days and the coefficient of variation consistent with that of SARS (mean over standard deviation) of 0.45, corresponding to $\alpha$=4.94 [36]. Under these assumptions we obtain the following posterior distributions shown in Figure S12.

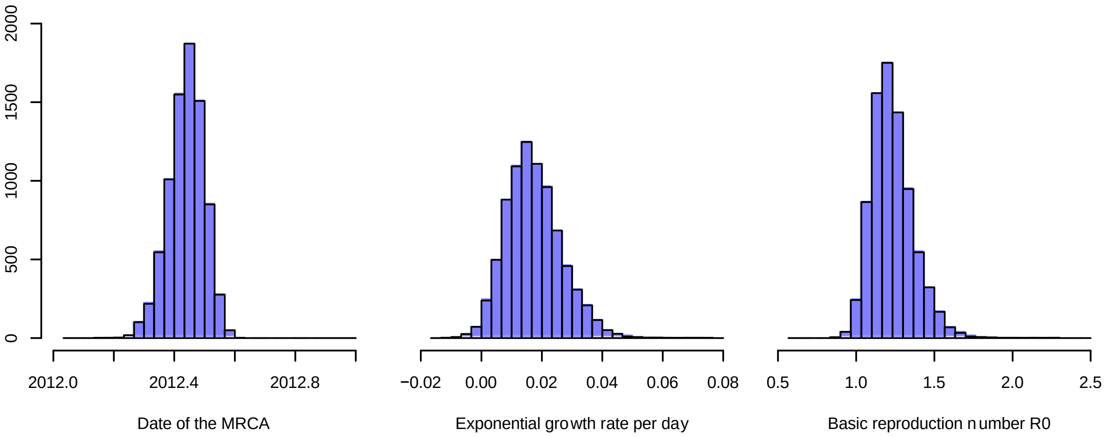


**Figure S12. Posterior distribution of the TMRCA, population growth rate and $R_0$.**

### 9.5.4 Number of infected individuals

We use two methods to estimate population sizes. Population sizes in BEAST, $N_B$, are reported in units of effective population size in coalescent time, i.e. the population size is $N_B = N_e \times T_g$ where $N_e$ is the effective population size and $T_g$ is the generation time. The effective population size $N_e$ is related to the census population size $N$ by a further transformation. Assuming a negative binomial offspring distribution and nearly neutral evolution, then the relationship between the effective population size and the actual population size is given by $N_1(t_c) = N_B R_0 (R_0 + k)/(kT_g)$ [37, 38], where $t_c$ is the current time. The population size at any other time is given by $N_1(t) = N_1(t_c) \exp[r(t - t_c)]$. In estimating $N_1$, we assume by default that $k = 1$. An alternative, and simpler, way to estimate the number infected is as $N_2(t) = \exp[r(t - t_0)]$ where $r$ is the growth rate, $t_0$ is the estimated TMRCA. Ideally, these estimates should be very similar if the epidemic has started from a single case and if estimates of $T_g$ and $k$ are accurate, but this consistency is not enforced in BEAST. We report both values. More specifically, we report the estimates of the cumulative number of people infected, i.e. $N_i(t_0)(\exp[r(t_s - t_0)] - 1)/(rT_g)$ for $i = 1,2$, where $t_a$ is the reported analysis time for this study (set as 8 August 2013).

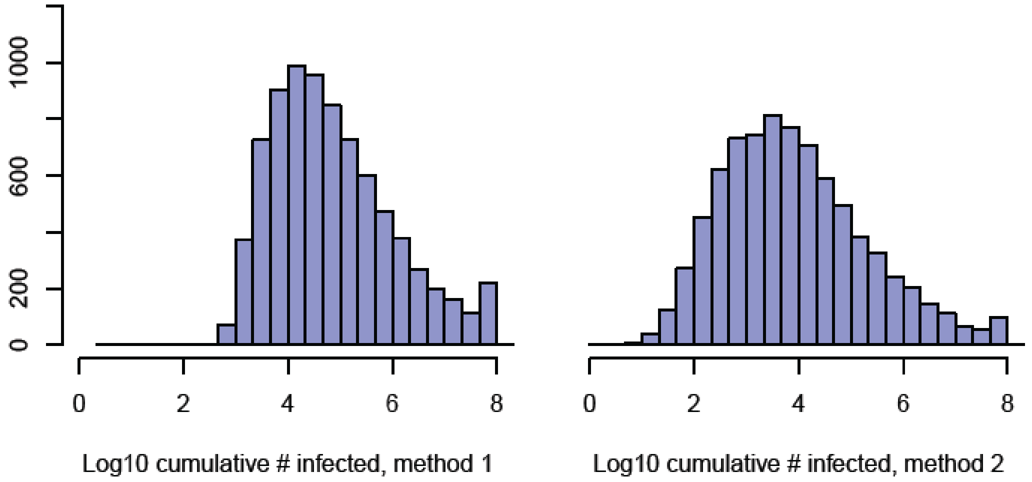


**Figure S13. Posterior distribution of the cumulative number of infections derived by the two methods described in the text.**

## 9.6 Sensitivity analyses

### 9.6.1 Sensitivity analysis to the choice of rooting

As mentioned above, there are two plausible rootings for the tree of all ten MERS-CoV viruses, which yield two different estimates of the clock rate of $5 \times 10^{-4}$ and $1.5 \times 10^{-3}$ per site per year, respectively. As no cases were reported prior to 2012, we judge the faster estimate to be somewhat more plausible, but neither can be ruled out. Here, we present results for the alternative substitution rate of $5 \times 10^{-4}$. The time-resolved MCC tree for the slower alternative ($5 \times 10^{-4}$) is shown in Figure S14.

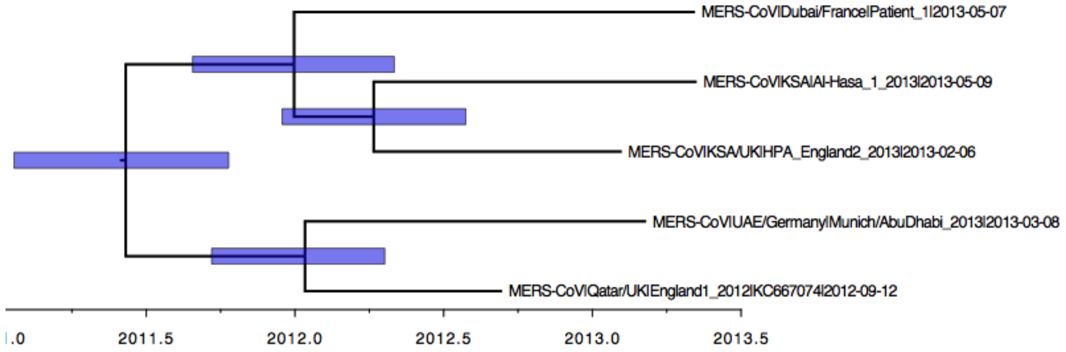


**Figure S14. Time resolved phylogeny of the recent clade of 5 samples assuming the slower clock rate** ($5 \times 10^{-4}$).

The corresponding posterior estimates of population parameters are shown in Figures S15 and S16.

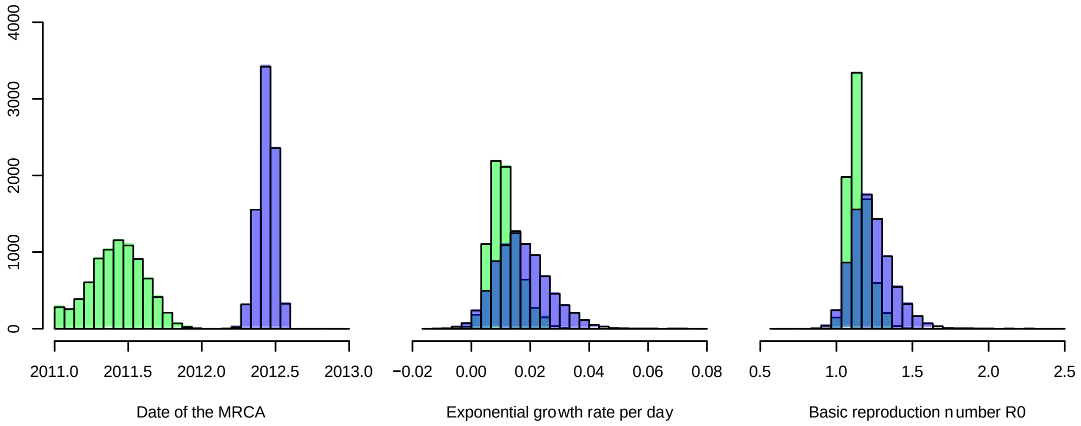


**Figure S15. Posterior distribution of the TMRCA, population growth rate and $R_0$ for the faster (blue) and slower (green) clock rates. The plots are semi-transparent, so darker colour represents overlap between the histograms.**

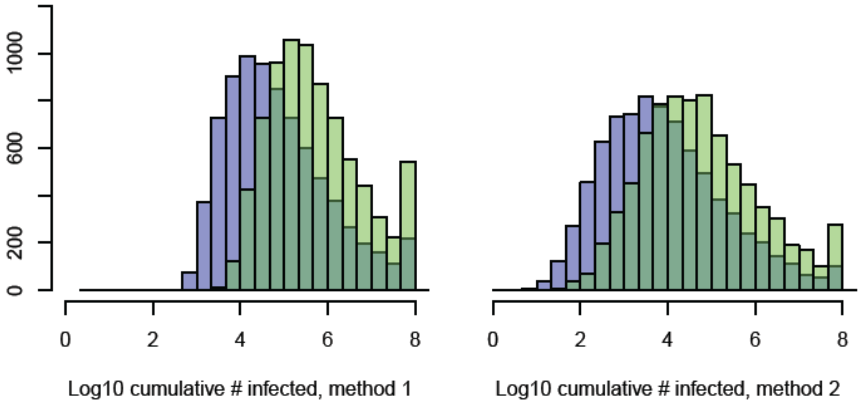


**Figure S16. Posterior distribution of the cumulative number of infections derived by the two methods described in the text for the faster (blue) and slower (green) clock rates. The plots are semi-transparent, so darker colour represents overlap between the histograms.**

### 9.6.2 Sensitivity to choice of clock rate

Table S14 summarises sensitivity of our estimates to the assumed clock rate.

**Table S14. Estimates of TMRCA, $R_0$ and the cumulative number of infections calculated by method 2 (see text) as a function of the assumed clock rate.**

| Clock rate | TMRCA (95% cr.i.) | $R_0$ (95% cr.i.) | Cumulative $N_2$ (IQR) |
|---|---|---|---|
| 5.0e-4 | 2011.43 (2011.03, 2011.76) | 1.14 (1.04, 1.31) | 39663 (5918, 363979) |
| 6.0e-4 | 2011.68 (2011.36, 2011.96) | 1.15 (1.04, 1.34) | 31598 (4789, 286669) |
| 7.0e-4 | 2011.88 (2011.59, 2012.11) | 1.17 (1.04, 1.4) | 27943 (3773, 308827) |
| 8.0e-4 | 2012.01 (2011.75, 2012.22) | 1.18 (1.04, 1.42) | 23080 (2920, 239478) |
| 9.0e-4 | 2012.12 (2011.91, 2012.3) | 1.2 (1.04, 1.49) | 25000 (3127, 272845) |
| 1.0e-3 | 2012.2 (2012, 2012.36) | 1.2 (1.04, 1.52) | 14730 (1954, 173209) |
| 1.1e-3 | 2012.27 (2012.09, 2012.42) | 1.22 (1.04, 1.51) | 16197 (2008, 166826) |
| 1.2e-3 | 2012.33 (2012.16, 2012.46) | 1.21 (1.03, 1.54) | 10006 (1332, 116413) |
| 1.3e-3 | 2012.37 (2012.22, 2012.49) | 1.21 (1.02, 1.53) | 6869 (989, 71249) |
| 1.4e-3 | 2012.41 (2012.27, 2012.52) | 1.22 (1.03, 1.54) | 8074 (1090, 76445) |
| 1.5e-3 | 2012.44 (2012.31, 2012.54) | 1.21 (1.02, 1.53) | 5636 (777, 55434) |
| 1.6e-3 | 2012.47 (2012.35, 2012.56) | 1.21 (1.02, 1.57) | 4322 (665, 37870) |
| 1.7e-3 | 2012.49 (2012.39, 2012.58) | 1.21 (1.01, 1.55) | 3392 (543, 34189) |
| 1.8e-3 | 2012.51 (2012.41, 2012.59) | 1.22 (1.01, 1.6) | 3724 (560, 38991) |
| 1.9e-3 | 2012.53 (2012.44, 2012.6) | 1.22 (1.02, 1.56) | 3176 (565, 29330) |
| 2.0e-3 | 2012.55 (2012.46, 2012.61) | 1.21 (1.01, 1.52) | 2676 (437, 23496) |
| 2.1e-3 | 2012.56 (2012.48, 2012.62) | 1.2 (1.01, 1.54) | 2099 (368, 16835) |
| 2.2e-3 | 2012.57 (2012.49, 2012.63) | 1.2 (1, 1.54) | 1967 (353, 17108) |
| 2.3e-3 | 2012.58 (2012.51, 2012.63) | 1.21 (1.01, 1.59) | 2006 (360, 16759) |
| 2.4e-3 | 2012.59 (2012.52, 2012.63) | 1.2 (1, 1.52) | 1600 (320, 11297) |
| 2.5e-3 | 2012.59 (2012.53, 2012.64) | 1.2 (1, 1.52) | 1526 (299, 10813) |

We show the inter-quartile range (IQR) for estimates of the number infected, as the 95% credibility intervals are extremely wide, reflecting the fact that the genetic analysis is only moderately informative with respect to this quantity.

### 9.6.3 Sensitivity to choice of generation time distribution

Table S15 summarises sensitivity of our estimates to the assumed mean generation time.

### 9.6.4 Table S15. Sensitivity of genetic estimates to mean generation time, $T_g$ , keeping coefficient of variation of the generation time distribution constant.

| $T_g$ (days) | $R_0$ (95% cr.i.) for clock rate = $1.5 \times 10^{-3}$ | $R_0$ (95% cr.i.) for clock rate =$5 \times 10^{-4}$ |
|---|---|---|
| 7 | 1.12 (1.01, 1.29) | 1.08 (1.02, 1.17) |
| 8 | 1.14 (1.01, 1.33) | 1.09 (1.03, 1.2) |
| 9 | 1.16 (1.02, 1.38) | 1.1 (1.03, 1.23) |
| 10 | 1.18 (1.02, 1.43) | 1.11 (1.03, 1.25) |
| 11 | 1.2 (1.02, 1.48) | 1.12 (1.04, 1.28) |
| 12 | 1.21 (1.02, 1.53) | 1.14 (1.04, 1.31) |
| 13 | 1.23 (1.02, 1.58) | 1.15 (1.04, 1.34) |
| 14 | 1.25 (1.02, 1.64) | 1.16 (1.05, 1.37) |
| 15 | 1.27 (1.03, 1.69) | 1.17 (1.05, 1.4) |
| 16 | 1.29 (1.03, 1.75) | 1.18 (1.05, 1.43) |

### *9.6.5* Sensitivity to choice of dispersion parameter *k*

The only estimated quantity that depends on the choice of dispersion parameter *k* is the cumulative population size estimated by method 1, $N_1$. With the default value *k*=1 (geometric offspring distribution), the posterior-median estimate is 45,976 (IQR: 9,419-359,662), while for *k*=0.16 (estimate for SARS from [25]) the estimate is 100,386 (IQR: 20,103-804,184). The increased estimate as *k* decreases, reflects the fact that lower *k* implies a greater fraction of people who infect no-one, and thus while infected do not contribute to the effective infectious population and hence observed viral evolution.

### 9.6.6 Sensitivity to choice of substitution model

All of the analyses were repeated with the HKY, GTR and SRD06 model instead of theTN93 model. Dependence on the choice of substitution model was found to be minimal (Table S16).

**Table S16. Estimates of TMRCA, $R_0$ and the cumulative number of infections calculated by method 2 (see text) for different substitution models and clock rates.**

| **Substitution model** | **= TN93** | | |
|---|---|---|---|
| **Clock rate** | **TMRCA (95% cr.i.)** | **$R_0$ (95% cr.i.)** | **Cumulative $N_2$ (IQR)** |
| 5.0e-4 | 2011.43 (2011.03, 2011.76) | 1.14 (1.04, 1.31) | 39663 (5918, 363979) |
| 1.0e-3 | 2012.2 (2012, 2012.36) | 1.2 (1.04, 1.52) | 14730 (1954, 173209) |
| 1.5e-3 | 2012.44 (2012.31, 2012.54) | 1.21 (1.02, 1.53) | 5636 (777, 55434) |
| 2.0e-3 | 2012.55 (2012.46, 2012.61) | 1.21 (1.01, 1.52) | 2676 (437, 23496) |
| 2.5e-3 | 2012.59 (2012.53, 2012.64) | 1.2 (1, 1.52) | 1526 (299, 10813) |
| **Substitution model** | **= HKY** | | |
| **Clock rate** | **TMRCA (95% cr.i.)** | **$R_0$ (95% cr.i.)** | **Cumulative $N_2$ (IQR)** |
| 5.0e-4 | 2011.43 (2011.03, 2011.76) | 1.13 (1.04, 1.28) | 32298 (5268, 281051) |
| 1.0e-3 | 2012.2 (2012, 2012.36) | 1.2 (1.04, 1.48) | 14482 (2029, 156319) |
| 1.5e-3 | 2012.44 (2012.32, 2012.54) | 1.21 (1.02, 1.56) | 5119 (790, 55458) |
| 2.0e-3 | 2012.55 (2012.46, 2012.61) | 1.2 (1.01, 1.51) | 2255 (408, 17926) |
| 2.5e-3 | 2012.59 (2012.53, 2012.64) | 1.2 (1, 1.54) | 1655 (316, 10825) |
| **Substitution model** | **= GTR** | | |
| **Clock rate** | **TMRCA (95% cr.i.)** | **$R_0$ (95% cr.i.)** | **Cumulative $N_2$ (IQR)** |
| 5.0e-4 | 2011.43 (2011.03, 2011.75) | 1.13 (1.04, 1.31) | 34548 (5454, 304665) |
| 1.0e-3 | 2012.2 (2012.01, 2012.36) | 1.2 (1.04, 1.5) | 14610 (2072, 164641) |
| 1.5e-3 | 2012.44 (2012.32, 2012.54) | 1.21 (1.02, 1.54) | 5049 (726, 51705) |
| 2.0e-3 | 2012.55 (2012.46, 2012.61) | 1.21 (1.01, 1.54) | 2655 (447, 23557) |
| 2.5e-3 | 2012.59 (2012.53, 2012.64) | 1.2 (1.01, 1.51) | 1722 (340, 1004) |
| **Substitution model** | **= SRD06** | | |
| **Clock rate** | **TMRCA (95% cr.i.)** | **$R_0$ (95% cr.i.)** | **Cumulative $N_2$ (IQR)** |
| 5.0e-4 | 2011.44 (2011.03, 2011.77) | 1.13 (1.04, 1.3) | 33568 (4947, 322555) |
| 1.0e-3 | 2012.21 (2012.02, 2012.37) | 1.21 (1.04, 1.5) | 17082 (2153, 237427) |
| 1.5e-3 | 2012.45 (2012.32, 2012.54) | 1.22 (1.03, 1.55) | 5597 (806, 56719) |
| 2.0e-3 | 2012.55 (2012.46, 2012.61) | 1.21 (1.01, 1.56) | 2310 (430, 19677) |
| 2.5e-3 | 2012.59 (2012.53, 2012.64) | 1.2 (1, 1.53) | 1491 (304, 10216) |

## 9.7 Update of the phylogenetic analysis, added in proof

After the review of this paper was complete, Cotten et al [39] published an extensive analysis of viral sequences collected from Saudi Arabia, and the sequences were made publicly available on GenBank. Here, we describe an updated version of our phylodynamic analysis which includes these latest sequences. In updating the analysis, we include only one representative of the 19 sequences collected from the large Al-Hasa outbreak, since the coalescent model used to estimate population dynamics assumes the sample being analysed is randomly drawn from the infected population. Because of the controversy surrounding the EMC/2012 and the Bisha_1_2012 sequences [39], we exclude both from our analysis.

The results of the revised systematic comparison of substitution models are given in Table S17.

**Table S17**

| Model | -LL | N Params | BIC |
|---|---|---|---|
| TN93+Γ | 41616 | 24 | 83479 |
| HKY+Γ | 41626 | 23 | 83488 |
| TN93+I+Γ | 41616 | 25 | 83489 |
| HKY+I+Γ | 41626 | 24 | 83498 |
| GTR+Γ | 41613 | 27 | 83503 |
| TN93 | 41636 | 23 | 83508 |
| GTR+I+Γ | 41613 | 28 | 83514 |
| HKY | 41646 | 22 | 83517 |
| TN93+I | 41636 | 24 | 83519 |
| HKY+I | 41646 | 23 | 83528 |
| GTR | 41636 | 26 | 83537 |
| GTR+I | 41636 | 27 | 83543 |

Increased number of parameters compared to Table S13 reflects more branch lengths to optimise in the estimation of the tree (# params = # substitution rates + #branch lengths). Compared to the previous analysis, the data are now better fitted with models with Gamma rate variation between sites, so the revised list of favoured models used in the analysis are the TN93+G, HKY+G, GTR+G. Here, we focus on the TN93+G model (with four rate categories for Gamma rate variation). The resulting maximum likelihood tree is shown in Figure S16.

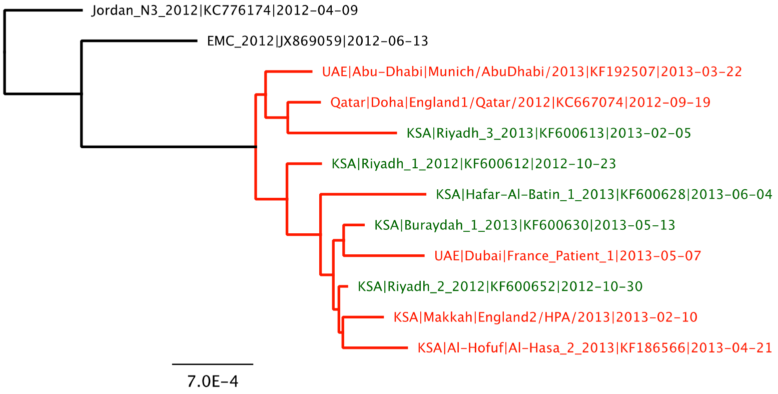


**Figure S16. Updated maximum phylogenetic tree, with new sequences shown in green and outgroup in black.**

The regression of root-to-tip distance on calendar time is shown in Figure S17, which shows a slight increase in the estimated clock rate, from $1.5 \times 10^{-3}$ per site per year for the previous analysis, to $1.8 \times 10^{-3}$ per site per year here. As in the earlier analysis, there is more than one well supported position for the root of the phylogeny, and the regression analysis of the alternative rooting is shown in Figure S18. This also shows an increase in the clock rate estimate, from $4.9 \times 10^{-4}$ previously to $9.4 \times 10^{-4}$ substitutions per site per year here, with a TMRCA in late 2010. We find below that this latter estimate is the most consistent with a new in-depth analysis of the ingroup.

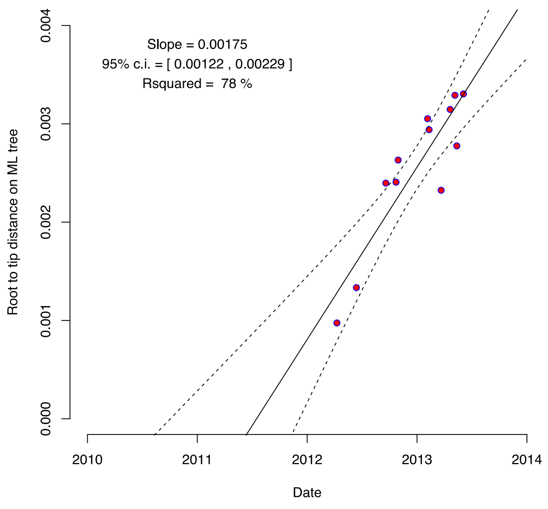


**Figure S17. Updated root-to-tip regression for the maximum likelihood phylogeny.**

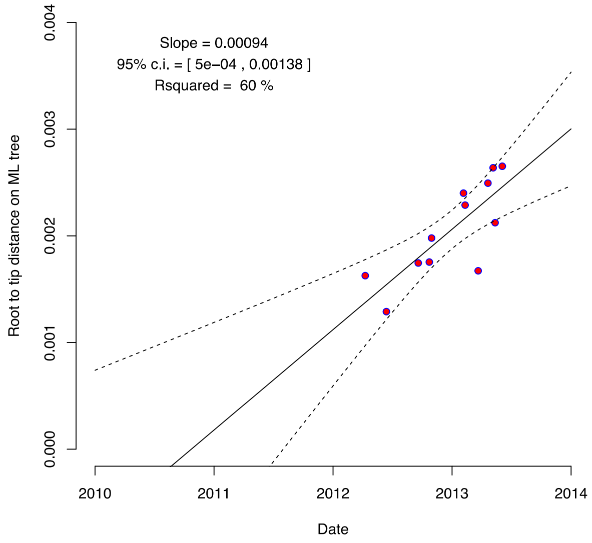


**Figure S18. Updated root-to-tip regression for the maximum likelihood phylogeny with alternative rooting, leading to a slower clock and earlier TMRCA.**

We updated the phylodynamic analysis with BEAST [34] to estimate the dated phylogeny and the changing population size, using identical similar assumptions to our previous analysis, with one important modification. As we had more data, we estimated the clock rate rather than assuming fixed values and varying over a fixed range. We assumed a strict clock, and chose an (improper) clock rate with uniform distribution on [0,∞[.

As before, convergence of the MCMC chains was excellent. We emphasise that it is not a

prior assumption that the infected population is growing, since the population size parameter and the rate of exponential change parameters are independent.

Figure S19 shows the estimates dated phylogeny drawn from the posterior distribution (maximum clade credibility).

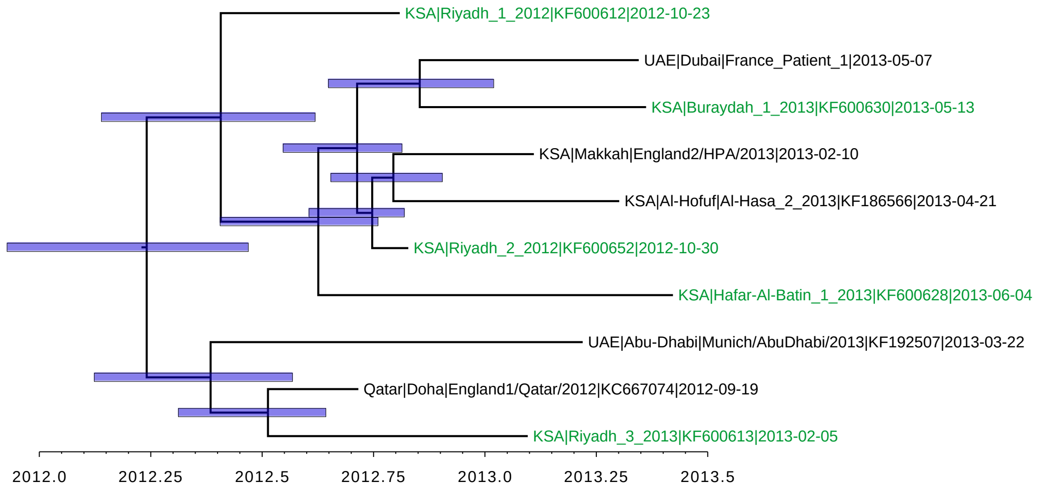


**Figure S19. Updated time resolved phylogeny of the recent clade of 10 samples. New sequences are shown in green.**

The estimates of the clock rate are presented in Figure S20. The posterior median estimate is $1.0 \times 10^{-3}$ substitutions per site per year (95% credibility interval $6.8 \times 10^{-4}$-$1.3 \times 10^{-3}$). This compares to estimates by Cotten et al [39], who find $6.3 \times 10^{-4}$ (95% credibility interval $1.4 \times 10^{-4}$-$1.1 \times 10^{-3}$). Differences in the central estimates may be the result of our inclusion of the new UAE|Dubai|France sequence which is relatively distant from the root, and exclusion of the controversial Bisha_1 sequence, which is relatively close to the root. These estimates are consistent, in that their credible intervals are highly overlapping.

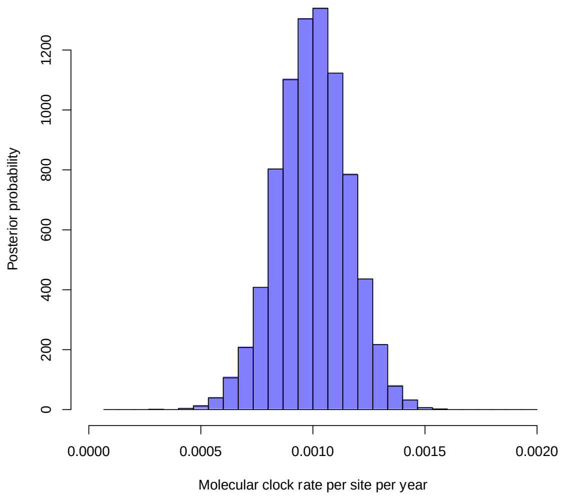

**Figure S20. Posterior distribution of the molecular clock rate, per site per year.**

The updated results from the population dynamics are shown in Figures S21 and S22 and Table S18. In all cases updated estimates are consistent with previous estimates, but with reduced uncertainty.

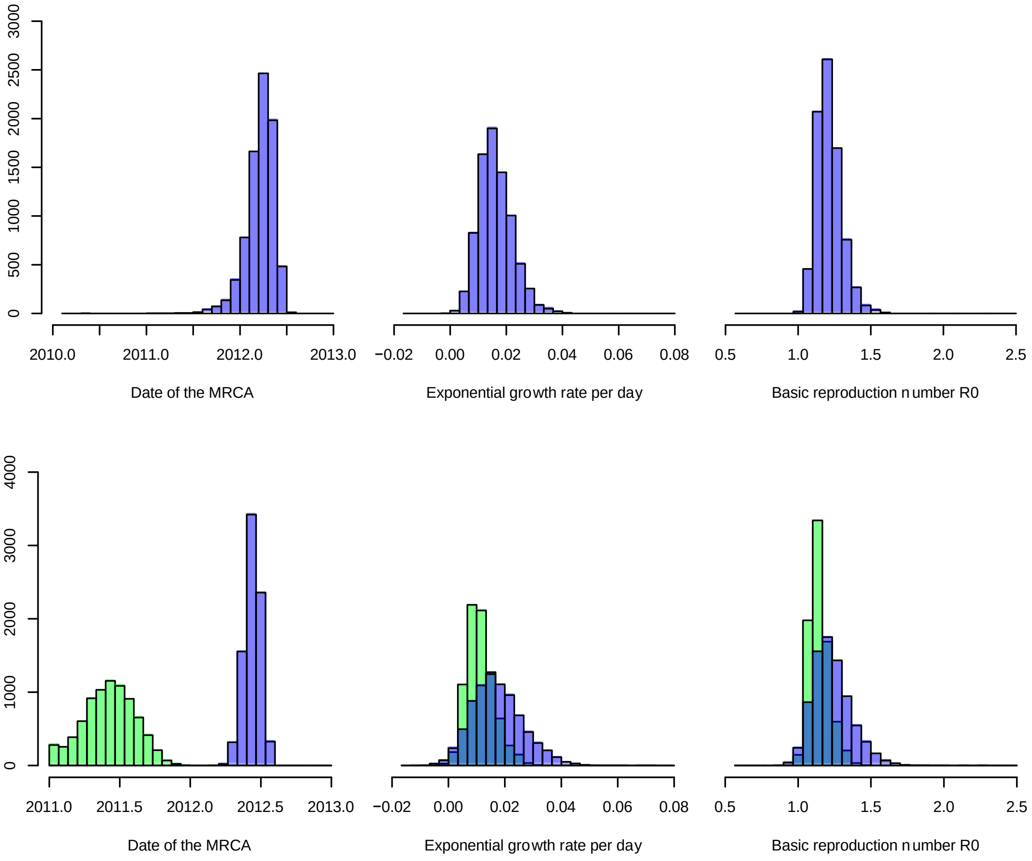


**Figure S21. Updated posterior distribution of the TMRCA, population growth rate and $R_0$. Upper panels: new results. Lower panels: old results, reproduced from figure S15.**

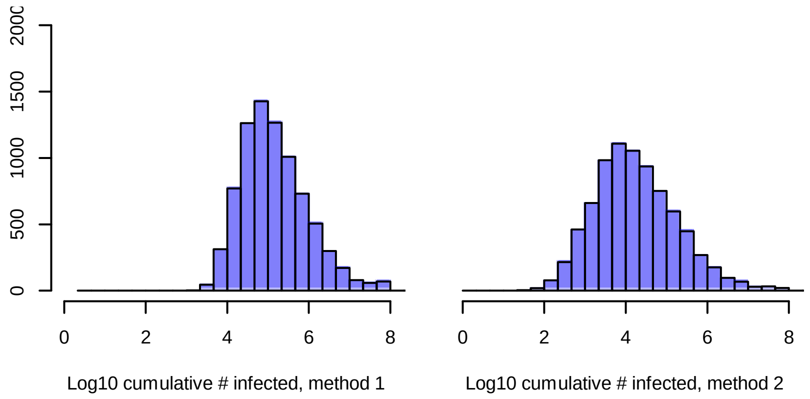


**Figure S22. Updated posterior distribution of the cumulative number infected (updated version of figure S16.**

**Table S18. Updated estimates of TMRCA, $R_0$ and the cumulative number of infections calculated by method 2 (see text) for different substitution models.**

| | | | |
|---|---|---|---|
| **Substitution model** | **= TN93+$\Gamma$** | | |
| **Clock rate (95% cr.i.)** | **TMRCA (95% cr.i.)** | **$R_0$ (95% cr.i.)** | **Cumulative $N_2$ (IQR)** |
| 1.0e-3 (6.8e-4-1.3e-3) | 2012.24 (2011.85, 2012.43) | 1.20 (1.08, 1.40) | 14340 (3419, 75006) |
| **Substitution model** | **= HKY+$\Gamma$** | | |
| **Clock rate (95% cr.i.)** | **TMRCA (95% cr.i.)** | **$R_0$ (95% cr.i.)** | **Cumulative $N_2$ (IQR)** |
| 1.0e-3 (6.9e-4-1.3e-3) | 2012.24 (2011.85, 2012.43) | 1.20 (1.08, 1.41) | 16098 (3820, 80538) |
| **Substitution model** | **= GTR+$\Gamma$** | | |
| **Clock rate (95% cr.i.)** | **TMRCA (95% cr.i.)** | **$R_0$ (95% cr.i.)** | **Cumulative $N_2$ (IQR)** |
| 1.0e-3 (6.9e-4-1.3e-3) | 2012.24 (2011.87, 2012.43) | 1.20 (1.08, 1.41) | 16029 (3613, 82795) |
| **Substitution model** | **= SRD06** | | |
| **Clock rate (95% cr.i.)** | **TMRCA (95% cr.i.)** | **$R_0$ (95% cr.i.)** | **Cumulative $N_2$ (IQR)** |
| 1.0e-3 (6.8e-4-1.3e-3) | 2012.24 (2011.86, 2012.43) | 1.21 (1.08, 1.41) | 17490 (3903, 94507) |

# 10 References


1. AlBarrak AM, Stephens GM, Hewson R, Memish ZA. Recovery from severe novel coronavirus infection. Saudi Med J. 2012; **33**(12): 1265-9.

2. Bermingham A, Chand MA, Brown CS, Aarons E, Tong C, Langrish C, et al. Severe respiratory illness caused by a novel coronavirus, in a patient transferred to the United Kingdom from the Middle East, September 2012. Euro surveillance : bulletin Europeen sur les maladies transmissibles = European communicable disease bulletin. 2012; **17**(40): pii=20290. Available online: http://www.eurosurveillance.org/ViewArticle.aspx?ArticleId=

3. Buchholz U, Müller MA, Nitsche A, Sanewski A, Wevering N, Bauer-Balci T, et al. Contact investigation of a case of human novel coronavirus infection treated in a German hospital, October-November 2012. Euro Surveill 2013;18(8):pii=20406 Available online: http://wwweurosurveillanceorg/ViewArticleaspx?ArticleId=20406 2013.

4. ECDC. Novel Coronavirus Updates Available at: http://www.ecdc.europa.eu/en/healthtopics/coronavirus-infections/whats-new/Pages/whats_new.aspx, Last Accessed 13 March 2013. 2012-2013.

5. Guery B, Poissy J, el Mansouf L, Séjourné C, Ettahar N, Lemaire X, et al. Clinical features and viral diagnosis of two cases of infection with Middle East Respiratory Syndrome coronavirus: a report of nosocomial transmission. The Lancet. 2013.

6. Hijawi B, Abdallat M, Sayaydeh A, Alqasrawi S, Haddadin A, Jaarour N, et al. Novel coronavirus infections in Jordan, April 2012: epidemiological findings from a retrospective investigation. Eastern Mediterranean Health Journal. 2013; **19**(Supplement 1): S12-S8.

7. Memish ZA, Alhakeem R, Stephens GM. Saudi Arabia and the emergence of a novel coronavirus. Eastern Mediterranean Health Journal. 2013; **19**(Supplement 1): S7-S11.

8. Memish ZA, Zumla AI, Al-Hakeem RF, Al-Rabeeah AA, Stephens GM. Family Cluster of Middle East Respiratory Syndrome Coronavirus Infections. NEJM. 2013; **Published 29 May 2013**: DOI: 10.1056/NEJMoa1303729.

9. Pebody RG, Chand MA, Thomas HL, Green HK, Boddington NL, Carvalho C, et al. The United Kingdom public health response to an imported laboratory confirmed case of a novel coronavirus in September 2012. Euro surveillance : bulletin Europeen sur les maladies transmissibles = European communicable disease bulletin. 2012; **17**(40): pii=20292. Available online: http://www.eurosurveillance.org/ViewArticle.aspx?ArticleId=

10. The Health Protection Agency (HPA) UK Novel Coronavirus Investigation team. Evidence of person-to-person transmission within a family cluster of novel coronavirus infections, United Kingdom, February 2013. Euro surveillance : bulletin Europeen sur les maladies transmissibles = European communicable disease bulletin. 2013; **18**(11): pii=20427. Available online: http://www.eurosurveillance.org/ViewArticle.aspx?ArticleId= T.
11. UK Health Protection Agency. Press Release: Third case of novel coronavirus infection identified in family cluster, 15 February 2013. Available at: http://www.hpa.org.uk/NewsCentre/NationalPressReleases/2013PressReleases/1302153rdcaseofcoronavirus/. Last Accessed 13 March 13. 2013.
12. UK Health Protection Agency. Press Release: Case of novel coronavirus identified in the UK. 11 February 2013 Available at: http://www.hpa.org.uk/NewsCentre/NationalPressReleases/2013PressReleases/130211statementonlatestcoronaviruspatient/ Last Accessed 13 March 13. 2013.
13. UK Health Protection Agency. Press Release: Further UK case of novel coronavirus. 13 February 2013. Available at: http://www.hpa.org.uk/NewsCentre/NationalPressReleases/2013PressReleases/130213statementonlatestcoronaviruspatient/. Last Accessed 13 March 13. 2013.
14. WHO. World Health Organization. Disease Outbreak News Novel Coronavirus. Available at: http://wwwwhoint/csr/don/archive/disease/coronavirus_infections/en/indexhtml Last Accessed 22July 2013. 2012-2013.
15. Zaki AM, van Boheemen S, Bestebroer TM, Osterhaus AD, Fouchier RA. Isolation of a novel coronavirus from a man with pneumonia in Saudi Arabia. The New England journal of medicine. 2012; **367**(19): 1814-20. doi: 10.056/NEJMoa1211721. Epub 2012 Oct 17.
16. Saudi Arabia Ministry of Health. Available at: http://www.moh.gov.sa/en/HealthAwareness/Corona/PressReleases/Pages/PressStatement-2013-05-29-001.aspx, Lat accessed 31 May 2013. 2012-2013.
17. ProMed. NOVEL CORONAVIRUS - SAUDI ARABIA (15): NEW CASE, Published Date: 2012-11-04 13:11:42. Available at: http://www.promedmail.org/direct.php?id=20121104.1391285. 2012.
18. ProMed. NOVEL CORONAVIRUS - EASTERN MEDITERRANEAN (17): SAUDI ARABIA Published Date: 2013-05-03 11:27:27 Available at: http://www.promedmail.org/direct.php?id=20130503.1688355. 2013.
19. ProMed. NOVEL CORONAVIRUS - EASTERN MEDITERRANEAN (18): SAUDI ARABIA. Published Date: 2013-05-05 18:49:21Available at: http://www.promedmail.org/direct.php?id=20130505.1693290. 2013.
20. ProMed. NOVEL CORONAVIRUS - EASTERN MEDITERRANEAN (21): SAUDI ARABIA.Published Date: 2013-05-09 10:10:04, Available at: http://www.promedmail.org/direct.php?id=20130509.1701527. 2013.
21. Assiri A, McGeer A, Perl TM, Price CS, Al Rabeeah AA, Cummings DAT, et al. Hospital Outbreak of Middle East Respiratory Syndrome Coronavirus. New England Journal of Medicine. 2013; **2013 Jun 19. [Epub ahead of print]**.
22. WHO. World Health Organization: MERS-CoV summary and literature update – as of 20 June 2013. Available at: http://www.who.int/csr/disease/coronavirus_infections/update_20130620/en/index.html. Last accessed 2 July 2013. 2013.
23. Omrani AS, Matin MA, Haddad Q, Al-Nakhli D, Memish ZA, Albarrak AM. A family cluster of Middle East Respiratory Syndrome Coronavirus infections related to a likely unrecognized asymptomatic or mild case. International journal of infectious diseases : IJID : official publication of the International Society for Infectious Diseases. 2013; **17**(9): e668-e72.

24. Saudi Arabia Ministry of Health. Media Statements. Corona. Available at: http://www.moh.gov.sa/en/HealthAwareness/Corona/Pages/PressStatements.aspx, last acessed 15 August 2013. 2013.
25. Lloyd-Smith JO, Schreiber SJ, Kopp PE, Getz WM. Superspreading and the effect of individual variation on disease emergence. Nature. 2005; **438**(7066): 355-9.
26. Nishiura H, Yan P, Sleeman CK, Mode CJ. Estimating the transmission potential of supercritical processes based on the final size distribution of minor outbreaks. J Theor Biol. 2012; **294**: 48-55.
27. Wallinga J, Teunis P. Different epidemic curves for severe acute respiratory syndrome reveal similar impacts of control measures. Am J Epidemiol. 2004; **160**(6): 509-16.
28. Andrieu C, Doucet A, Holenstein R. Particle Markov chain Monte Carlo methods. Journal of the Royal Statistical Society: Series B (Statistical Methodology). 2010; **72**(3): 269-342.
29. Spiegelhalter DJ, Best NG, Carlin BR, van der Linde A. Bayesian measures of model complexity and fit. J Roy Stat Soc B. 2002; **64**: 583-616.
30. Cotten M, Lam TT, Watson SJ, Palser A, Petrova V, Grant P, et al. Full-genome deep sequencing and phylogenetic analysis of novel human betacoronavirus. Emerging infectious diseases. 2013; **19**(5): 736-42B. doi: 10.3201/eid1905.130057.
31. van Boheemen S, de Graaf M, Lauber C, Bestebroer TM, Raj VS, Zaki AM, et al. Genomic characterization of a newly discovered coronavirus associated with acute respiratory distress syndrome in humans. mBio. 2012; **3**(6).
32. Salemi M, Fitch WM, Ciccozzi M, Ruiz-Alvarez MJ, Rezza G, Lewis MJ. Severe acute respiratory syndrome coronavirus sequence characteristics and evolutionary rate estimate from maximum likelihood analysis. Journal of virology. 2004; **78**(3): 1602-3.
33. Zhao Z, Li H, Wu X, Y. Z, Zhang K, Zhang YP, et al. Moderate mutation rate in the SARS coronavirus genome and its implications. BMC Evol Biol. 2004; **4**: 21.
34. Drummond AJ, Rambaut A. BEAST: Bayesian evolutionary analysis by sampling trees. BMC Evolutionary Biology 2007; **7**: 214.
35. Wallinga J, Lipsitch M. How generation intervals shape the relationship between growth rates and reproductive numbers. Proceedings of the Royal Society B: Biological Sciences. 2007; **274**(1609): 599-604.
36. Lipsitch M, Cohen T, Cooper B, Robins JM, Ma S, James L, et al. Transmission dynamics and control of severe acute respiratory syndrome. Science. 2003; **300**(5627): 1966-70. Epub 2003 May 23.
37. Magiorkinis G, Sypsa V, Magiorkinis E, Paraskevis D, Katsoulidou A, R. B, et al. Integrating phylodynamics and epidemiology to estimate transmission diversity in viral epidemics. PLoS Comput Biol. 2013; **9**(1): e1002876. doi: 10.1371/journal.pcbi.. Epub 2013 Jan 31.
38. de Silva E, Ferguson NM, Fraser C. Inferring pandemic growth rates from sequence data. J R Soc Interface. 2012; **9**(73): 1797-808.
39. Cotten M, Watson SJ, Kellam P, Al-Rabeeah AA, Makhdoom HQ, Assiri A, et al. Transmission and evolution of the Middle East respiratory syndrome coronavirus in Saudi Arabia: a descriptive genomic study. Lancet. 2013.
